# Effect of Non-*Saccharomyces* Yeasts Derived from Traditional Fermented Foods on Beer Fermentation Characteristics and Flavor Profiles

**DOI:** 10.3390/foods14081395

**Published:** 2025-04-17

**Authors:** Yanlin Ma, Liangyu Liu, Guanhui Hu, Shuyi Wang, Lei Shan, Jingyu Chen

**Affiliations:** 1 Key Laboratory of Food Bioengineering (China National Light Industry), College of Food Science and Nutritional Engineering, China Agricultural University, Beijing 100083, China; b20203060476@cau.edu.cn (Y.M.); huhuzi2002@163.com (G.H.); wshuyi1023@163.com (S.W.); 2 Kweichow Moutai Co., Ltd., Zunyi 564501, China; aleliuliangyu@163.com; 3 Department of Food Science and Nutrition, University of Minnesota, Saint Paul, MN 55108, USA; shanx057@umn.edu; 4 Sichuan Advanced Agricultural & Industrial Institute, China Agricultural University, Chengdu 611430, China

**Keywords:** screening, *Saccharomycopsis fibuligera*, non-*Saccharomyces* yeast, beer, flavor

## Abstract

In recent years, numerous studies have demonstrated that non-*Saccharomyces* yeasts hold potential for industrial application and aroma generation during fermentation. Non-*Saccharomyces* wild yeasts can be important tools in the development of new products, and the objective of this work was to obtain and characterize novel yeast isolates for their ability to produce beer. Traditional fermented beverages serve as a vital source of yeast strains that can exhibit unique characteristics during the brewing process. Thus, 22 strains of *Saccharomycopsis fibuligera* were isolated from traditional fermented foods in this work. Subsequently, through primary and secondary screening, *S. fibuligera* G02 was identified as a promising candidate for beer brewing, attributed to its advantageous physiological traits and notable potential for beer production. Headspace solid-phase microextraction (HS-SPME) combined with gas chromatography–mass spectrometry (GC–MS) was employed to analyze the volatile flavor substances in beer fermented using the *S. fibuligera* G02 strain. Chemometric analysis revealed that *S. fibuligera* G02 had a unique influence on beer aroma. Accordingly, isoamyl alcohol, phenyl-1-ethanol, ethyl acetate, isoamyl acetate, and 4-ethyl guaiacol (4EG) were the key aroma components of *S. fibuligera* G02. This work provides useful insights into the non-*Saccharomyces* yeasts to reference the targeted improvement of beer aroma.

## 1. Introduction

Beer, one of the oldest fermented beverages, is produced by yeast fermentation with wort and certain adjuncts such as hops [1]. The aroma and flavor of beer primarily derive from esters, higher alcohols, and phenolic compounds that together create its distinctive profile, making beer one of the most popular beverages worldwide [2]. Higher alcohols such as isoamyl alcohol and phenyl ethyl alcohol, along with their ester derivatives, isoamyl acetate and 2-phenyl ethyl acetate, are key aroma compounds primarily responsible for the floral and fruity notes valued in many beer styles [3]. Conversely, certain volatiles, including phenolic compounds, are generally considered undesirable due to their tendency to produce off-flavors such as rancidity or buttery notes [4]. However, concentrations of these compounds vary greatly within different beer styles. The influence of 4-vinyl guaiacol (4VG) with a “clove-like” descriptor is essential in certain traditional ales [3]. The compound 4-ethyl guaiacol (4EG) with “spicy”, “clove-like”, and “vanilla” descriptors is present in spontaneous fermentations of Gueuze and Lambic beers, in which it contributes to the typical sourness and unique complex flavor profile [5].

Traditionally, the genus *Saccharomyces*, including *Saccharomyces cerevisiae* (ale beer) and *Saccharomyces pastorianus* (lager beer), has been widely utilized in industrially produced beer due to the distinctive advantages [6]. However, limited microbial diversity during fermentation reduces the complexity of the sensory profile of beer [7]. Numerous studies have suggested that several non-*Saccharomyces* species are gaining attention due to their contribution to aroma complexity and the increased yield of desirable compounds [8]. From this perspective, enhancing yeasts with diverse flavor characteristics is of prime importance to satisfy global consumer trends for new and natural beverages [9]. A potential alternative to overcome this challenge is to carry out pure fermentation using non-*Saccharomyces* yeasts such as *Lachancea thermotolerans*, *Pichia kluyveri*, and *Hanseniaspora* that have been specifically selected for their performance and distinctive fruity characteristics [10]. These efforts also have predominantly resulted in the production of non-alcoholic beer (0.00–0.50% alcohol by volume, ABV) or low-alcohol beer (0.60–3.50% ABV). Mix fermentation between *Saccharomyces* and non-*Saccharomyces* yeasts represents an additional strategy for enhancing aroma. Typically, *Saccharomyces* species exhibit robust fermentation performance but produce only low concentrations of certain desirable aroma compounds [11]. In contrast, while non-*Saccharomyces* yeasts tend to be less efficient in fermentation or yield lower alcohol contents, they are capable of generating higher concentrations of novel flavor compounds [6,12,13]. By strategically combining *Saccharomyces* and non-*saccharomyces* yeasts, brewers can leverage their strengths, optimize fermentation efficiency, and enhance aroma profiles [10]. Mixed fermentation of *S. cerevisiae* with non-*Saccharomyces* yeasts such as *Metschnikowia pulcherrima*, *Schizosaccharomyces pombe*, *Lachancea thermotolerans*, *Torulaspora delbrueckii,* and various species of *Hanseniaspora* and *Pichia* has yielded highly promising results [14,15].

Exploring new habitats of microorganisms as potential sources of beer brewing and culture and phenotypically characterizing them holds promise for unveiling novel metabolic capabilities of microbial foods, including new flavors, textures, and nutritionally valuable microbes [16]. Currently, the exploration of new applications for non-*Saccharomyces* yeasts isolated from traditional fermented foods to diversify beer aroma profiles is being actively pursued [12,17,18]. Traditional beverages often contain highly complex yet resilient communities of microorganisms, in contrast to modern starter cultures that typically require controlled growth conditions to thrive [16,19]. These microorganisms of traditional fermented foods possess the potential to be used as starter cultures for the fermentation of traditional beverages and as innovative alternatives for producing various alcoholic beverages [20]. Little is known regarding the possible applications of these strains in beer production, and the discovery of new strains with industrial potential as substrates is highly likely [17]. Baijiu, a traditional fermented alcoholic beverage that originated in China, is made from sorghum, wheat, and rice through a complex fermentation process using natural mixed-culture starters (*Daqu*) that are the principal substrate for producing these beverages, and it is subsequently distilled to produce Baijiu [21]. The Baijiu production process harbors diverse microbiomes, with microbial communities playing a significant role in shaping the taste and flavor characteristics of the liquor [22].

The Baijiu production process has emerged as a novel and promising source of new yeasts for the brewing industry [23]. Nevertheless, the use of non-*Saccharomyces* yeasts from this source for beer brewing has not been trialed to date. For this reason, in this study, 24 strains of yeast were identified and isolated from Baijiu *Daqu* and fermentation grain. Primary screening and two-stage rescreening were performed to identify yeast strains with promising technological characterization. Here, one such strain, *Saccharomycopsis fibuligera* G02, could be propagated using traditional yeast growth media, tolerated various stressors associated with beer fermentation, and produced beer with a pleasant organoleptic profile. Subsequently, the fermentation parameters of the screened yeast strain *S. fibuligera* G02 were optimized, with a focus on temperature, inoculation rate, and International Bitterness Units (IBU) level [24,25,26]. Finally, the effect of *Saccharomycopsis fibuligera* inoculum during beer fermentation and sensory quality were evaluated using headspace solid-phase microextraction (HS-SPME) and analyzed using gas chromatography–mass spectrometry (GC–MS) combined with chemometrics.

## 2. Materials and Methods

### 2.1. Materials

*Daqu* (a starting material used for Baijiu production) and fermented grains (used for Baijiu distillation) were collected from different Baijiu-making enterprises in Xinghua Village (Shanxi Province), Bozhou City (Anhui Province), Luzhou City (Sichuan Province), and Maotai Town (Guizhou Province) for the screening of yeast strains (see Figure S1 and Table S1).

In this study, the following yeast strains were used as controls: *Saccharomyces cerevisiae* WB-06 (Fermentis, Lesaffre, France), *Saccharomyces cerevisiae* US-05 (Fermentis, Belgium), *Saccharomyces cerevisiae* Saison, and *Saccharomyces fibuligera* B0 (CGMCC No. 2.5608). The *S. fibuligera* B0 strain was isolated from a light-flavor Baijiu distillery in Beijing, China, and obtained from the China General Microbiological Culture Collection Center (CGMCC).

### 2.2. Methods

#### 2.2.1. Isolation of Yeasts from *Daqu* and Fermented Grains

An amount of 25 g of each sample was aseptically collected and individually added to 200 mL of sterile 0.9% saline solution. The mixture was centrifuged at 120 rpm for 30 min using a refrigerated benchtop centrifuge (Sigma-Aldrich, Steinheim, Germany) at room temperature [27,28]. A volume of 10 μL of each sample was used for microbial isolation on a YPD plate supplemented with chloramphenicol (100 mg/L) and then enrichment cultured at 30 °C for 48 h [12]. Isolates were purified by streak plating, cultured in 10 mL of YPD medium, and then visualized by phase-contrast microscopy (Motic Co., Hangzhou, China) at 1000× magnification to assess their morphology and purity [29].

#### 2.2.2. Strain Identification

A colony of each isolate was cultivated in liquid YPD medium, and the cell pellet from 200 μL of culture was used for genomic DNA (gDNA) extraction. Next, 0.5 μL of genomic DNA (gDNA) was used as the template for PCR amplification of the variable domain (D1/D2) of the 26S rRNA gene using primers NL1 (5′-GCA-TATCAATAAGCGGAGGAAAAG-3′) and NL4 (5′-GGTCCGTGTTTCAAGACGG-3′) [30], resulting in PCR products of approximately ~600 bp. Then, the purified polymerase chain reaction (PCR) products of each strain were sequenced by Tsingke Biotechnology Co., Ltd. (Beijing, China). BLAST software (Molecular Evolutionary Genetics Analysis) v.10.1.7 (https://www.megasoftware.net) for alignment, searches were performed using the NCBI (http://www.ncbi.nlm.nih.gov/blast, and a phylogenetic tree was constructed using the neighbor-joining method in MEGA 6. Phylogeny and phylogenetic relationships were analyzed based on the phylogenetic tree. The identified yeasts were mixed with 30% glycerol and stored at −80 °C to establish a culture bank.

#### 2.2.3. Screening of the Non-*Saccharomyces* Yeast Strains

1. Primary screening: growth ability in medium with maltose as the sole carbon source

Yeast strains were inoculated into 5 mL of YPD medium (10 g L^−1^ of yeast extract, 20 g L^−1^ of peptone, and 20 g L^−1^ of D-glucose) and incubated overnight at 30 °C with agitation (Shanghai Yiheng Scientific Instruments Co., Shanghai, China). The optical density (OD_600_) of the cultures was measured using a UNIC UV4802 UV/Vis spectrophotometer, and the cultures were then diluted with sterile water to prepare an initial culture (OD_600_ = 0.5) [29]. Strains were then grown in a synthetic medium containing maltose as the sole carbon source with 1% inoculum. An uninoculated medium served as a blank (negative control). All experiments were performed in triplicate for 72 h at 30 °C. The synthetic medium consisted of MgSO_4_·7H_2_O (1 g L^−1^), KH_2_PO_4_ (1g L^−1^), maltose (33.7 g L^−1^), NaCl (1 g L^−1^), 10 g L^−1^ of yeast extract, and peptone (20 g L^−1^), and was sterilized at 115 °C for 30 min [31].

2. First round of rescreening: evaluation of physiological characteristics

The variables tested are relevant to both standard brewing practices (e.g., temperature adaptability and osmotic stress tolerance) and industrial fermentation processes (e.g., salt tolerance) [29]. Temperature trials were conducted in YPD medium at 10 °C, 20 °C, 30 °C, and 37 °C to assess tolerance to different temperatures for growth, while maintaining the natural pH. Salinity tolerance tests were performed using YPD medium supplemented with 1%, 5%, 10%, or 20% (*w*/*v*) NaCl. Growth in different pH conditions of the YPD medium was evaluated at pH 2.5, 3.5, 5.0, and 8.0 (adjusted by 1 M HCl or NaOH) before the inoculation of yeast. Ethanol tolerance was assessed in the YPD medium supplemented with 5, 10, 15, or 20% (*v*/*v*) ethanol. Growth ability was assessed on a YP medium (10 g L^−1^ of yeast extract and 20 g L^−1^ of peptone) with various carbon sources (2% *w*/*v* glucose, maltose, sucrose, or galactose). Osmotic stress tolerance was evaluated using a YP medium supplemented with 10% and 20% *w*/*v* glucose and maltose. The final OD_600nm_ of all pre-cultures was 0.6 with a 1% inoculation size. A non-inoculated medium served as a blank (negative control). Trials were performed at 30 °C, except for temperature trials. Experiments were conducted in triplicate in 96-well plates for 48 h, which were sealed to prevent contamination (Thermo Fisher Scientific, Waltham, MA, USA) [26].

3. Second round of rescreening: Beer fermentation test

The artisanal wort approach, as described by Tan et al. [32], was modified appropriately. Worts were boiled using hops from Tsingtao Flower, sourced in Gansu, and then adjusted to a pH of 5.5 with 0.2% lactic acid and had a characterized gravity of 13 °Plato (°P).

Cultures of selected yeasts were inoculated into 100 mL YPD medium and shaken at 180 rpm (Beijing Donglian Haer Instrument Manufacturing Co., Ltd., Beijing, China) for 48 h to obtain the starter culture at 30 °C. Viable yeast cells (CFU/mL) were counted by a Countstar^®^ automated cell counter (Count Star, Shanghai, China) [33]. The selected yeasts were then inoculated with a pitching rate of 1 × 10^7^ cells/mL (corresponding to 7 log CFU/ mL) in 500 mL Erlenmeyer flasks, with an air-lock to release CO_2,_ containing 250 mL of artisanal wort. Fermentation was carried out at the optimal temperature for the yeast strain at 30° C for 6 d [32]. During fermentation, visible fermentation activity (e.g., gas release through the air-lock) and biofilm formation on the liquid surface were observed. After 7 days, samples were collected for sensory analysis.

Sensory evaluation was performed using a validated questionnaire administered to a panel of 23 assessors (13 females and 10 males, aged 21–22 years) recruited from the College of Food Science and Nutrition Engineering. Odor is the perception of volatile compounds outside the mouth (orthonasal odor). Sensory attributes included general descriptors (aroma intensity, fullness, and balance) and specific aromas (flowery, fruity, herbal, malt, and clove) rated from 0 to 7. Panelists were trained for two–three weeks to familiarize themselves with aroma descriptors, first learning beer-related aromas and then intensity scales. Standard reference samples were assessed until all standards were correctly identified. The samples were evaluated based on descriptors, with scores ranging from 0 to 7. Sensory analysis was conducted at room temperature (approximately 23 °C) in a distraction-free room, with each panelist in separate evaluation spaces. Samples were labeled with three-digit random numbers and maintained at 12 ± 1 °C [34]. Sensory evaluation scores and physicochemical parameters of fermented beer are presented in Supplementary Table S2, respectively. Each participant was informed of and agreed to the principles of the analysis. The data is confidential and will not be used without their knowledge. Participation in sensory evaluation of products was voluntary. 

#### 2.2.4. Fermentation Optimization of *S. fibuligera* G02

Artisanal no-hopped wort was prepared, with the pH adjusted to 5.5 using 20% lactic acid, and then diluted to 12 or 13 °P (as described in Section 2.2.3) [32]. Fermentation parameter optimization was carried out in EBC tubes (Figure S3), as described by Yin et al. [33].

1. Fermentation temperature optimization

The *S. fibuligera* G02 strain was inoculated at 1 × 10^6^ cells/mL into 13 °P no-hopped wort. The fermentation performance of the *S. fibuligera* G02 strain was evaluated at 22 °C, 26 °C, and 30 °C. Control strains were *S. cerevisiae* US-05 at 22 °C, *S. cerevisiae* WB-06 at 26 °C, and *S. cerevisiae* Saison at 30 °C. These commercial *S. cerevisiae* strains were rehydrated following the manufacturer’s recommendations, plated on YPD agar, and then incubated at 30 °C. Yeast culture activation and expansion were described in Section 2.2.3.

2. Optimization of initial inoculation rates

The *S. fibuligera* G02 strain was inoculated at either 1 × 10^6^ cells/mL or 1 × 10^7^ cells/mL rates into 13 °P no-hopped wort at 30 °C.

3. Optimizing initial concentrations of iso-α-acids of wort

The *S. fibuligera* G02 strain was inoculated at 1 × 10⁷ cells/mL. The fermentation experiment was carried out at 30 °C using 12 °P wort with iso-α-acid concentrations of 0, 10, and 25 IBU.

4. Validation of optimized fermentation parameters

The *S. fibuligera* G02 strain was inoculated at 1 × 10^7^ cells/mL into 13°P wort of 10 IBU and fermented at 26 °C for 7 d, with *S. cerevisiae* WB-06 as a control strain [12,34]. After 7 d, the alcohol content was measured using an Anton Paar analyzer (Anton Paar GmbH, Graz, Austria) [35].

5. Fermentation analysis.

During fermentation, samples were collected from each flask every 24 h for downstream analysis and analyzed in triplicate. The suspended yeast cells (CFU/mL) and wort gravity (°Plato, °P) were measured using a Countstar^®^ automated cell counter (Count Star, China) and a DMA 35 N (Anton Paar GmbH, Austria), respectively [12,35]. Fermentability, expressed as apparent fermentation degree, was calculated using the following formula:

Apparent fermentation degree (ADF, %) = [(original wort gravity − final gravity)/original wort gravity)] × 100%(1)

#### 2.2.5. Micro-Fermentation

1. Beer fermentation set-up

Fermentis WB-06 yeast served as a control in both single-strain and dual-strain sequential fermentations. The yeast strains *S. cerevisiae* NX02, *S. fibuligera* B0, and *S. fibuligera* G02 were used for single-strain fermentations, as well as in dual-strain sequential fermentations. The activation and expansion of yeast cultures are described in Section 2.2.3. For single-strain fermentations, each yeast strain was inoculated at rates of 1 × 10^7^ cells/mL. In dual-strain sequential fermentations, strains G02 or B0 were initially inoculated at a rate of 1 × 10^7^ cells/mL, followed by the inoculation of strains NX02 and WB-06 at the same rate after 48 h (refer to Figure S4 for the fermentation strategy). Primary fermentation was carried out in 500 mL Erlenmeyer flasks containing 250 mL of 12 °P wort medium at 26 °C for 7 d.

2. Fermentation analysis.

Yeast count samples were collected from each flask every 24 h and analyzed in triplicate. Colonies of *S. fibuligera* strains and *S. cerevisiae* strains were visually distinguishable due to their colony morphology characteristics (Figure S5), allowing for the monitoring of population dynamics during dual-strain sequential fermentation. Yeast count was determined by plating 100 µL of beer samples onto YPD agar supplemented with 100 mg L^−1^ chloramphenicol [12]. After 7 d, the apparent degree of fermentation (ADF) of each sample was calculated as described in Section 2.2.4.

#### 2.2.6. Volatile Compounds Analysis

1. HS-SPME

Beer samples were analyzed using headspace solid-phase microextraction (HS-SPME). A 5 mL sample was placed in a 15 mL bottle with 10 ppm of octan-1-ol (1 g L^−1^ in ethanol) as an internal standard and 0.5 g NaCl. The mixture was incubated at 45 °C for 20 min. SPME used a 2 cm fiber coated with 50/30 µm Divinylbenzene/Carboxen on PDMS for 40 min, and this was followed by gas chromatography at 270 °C for 5 min and GC-MS analysis.

2. Chromatographic conditions for GC–MS analysis

The analysis was performed using an Agilent 7890B GC-MS system (Agilent Technologies, USA) equipped with an HP-DBWAX column. Helium was used as the carrier gas. The flow rate was 1.0 mL/min with a splitless injection, and the inlet temperature was 250 °C. The initial temperature was held at 35 °C for 4 min, then ramped to 150 °C at 20 °C/min, further increased to 230 °C at 4 °C/min, and held at 230 °C for 10 min.

An electron ionization (70 eV, 170 °C) source was used, with a scanning range of 40 to 600 *m*/*z*. The temperature was initially set to 40 °C for 5 min and then increased to 230 °C at a rate of 6 °C/min. Retention times were matched to the NIST14 database for quantification using the following formula:

C1 = C2 × (A1/A2)(2)

Here, C1 is the concentration of the component (mg L^−1^), C2 is the internal standard concentration (mg L^−1^), A1 is the measured peak area, and A2 is the internal standard peak area.

#### 2.2.7. Statistics

All experiments were performed in triplicate, and results are reported as mean ± standard deviation. Significance was determined at *p* < 0.05, using *t*-tests and one-way ANOVA using SPSS (version 19.0). Principal component analysis (PCA) was conducted to analyze the first round of rescreening of yeast strains, with a focus on evaluating their physiological characteristics. Additionally, PCA was employed to differentiate among the average levels of various volatile compounds and microfermentation activities during the fermentation process. This analysis utilized the ‘pheatmap’, ‘viridis’, ‘factoextra’, and ‘ggplot2’ packages in R 4.4.1 software.

## 3. Results and Discussion

### 3.1. The Isolation of the Yeasts from Baijiu Daqu and Fermented Grains

A total of 24 yeast strains isolated from *Daqu* and fermented grain samples were identified by 26S rRNA gene sequencing and subjected to strain-level phylogenetic analysis. (Figure S2). The results, showing similarities to sequences in the GenBank database, are detailed in Table 1. The identification revealed that these strains belong to two distinct genera: *Saccharomycopsis* and *Saccharomyces*. Non-*Saccharomyces* yeasts have recently gained popularity among brewers for their unique characteristics and diverse substrate assimilation patterns [36]. Evidence suggests that cross-system microbial applications are advantageous [10,16]. Yeasts from other food systems such as wine and honey have exhibited potential for beer brewing applications [12]. Baijiu microbiomes, along with their metabolites, exhibited flavor-related and geography-dependent characteristics [37]. Here, 24 yeast strains were isolated from samples collected at four different locations in China, spanning a latitude range of 27 °N to 39 °N (see Figure S1). However, the NX samples hardly isolated any *Saccharomycopsis* strains in this study, a finding that is consistent with other studies [38] (see Table 1; Table S1).

### 3.2. Screening of the Non-Saccharomyces Yeast Strains

#### 3.2.1. Primary Screening

**Figure 1:**
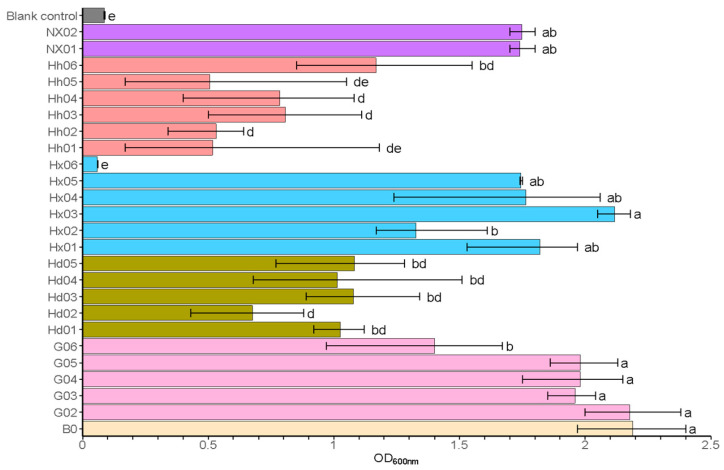
Primary screening: growth ability in medium with maltose as the sole carbon source. Data are presented as the final OD_600nm_ of the cultures. Error bars represent the standard deviation. Statistical differences were calculated using one-way ANOVA followed by Tukey’s test; different letters indicate statistically significant differences (*p* < 0.05). The uninoculated control represents the medium without inoculation (negative control).

Fermentable sugars, including glucose, maltose, and maltotriose, are essential nutrients for yeast, which they consume for cell growth, reproduction, and to enhance the quality of the final beer product [3,39]. Maltose stands out as the predominant disaccharide in both beer worts and the final products, and its utilization is critical in determining the characteristics of the beer [39]. As presented in Figure 1, isolates from light-flavor *Daqu* (Hh) and sauce-flavor *Daqu* (Hd) exhibited poor growth (OD_600_ ≤ 1.0). However, most *S. fibuligera* strains (G02-G06) from sauce-flavored fermented grain and most *S. fibuligera* strains (Hx01, Hx03, Hx04, and Hx05) from light-flavored *Daqu* (HX) exhibited strong growth (OD_600_ > 1.5). Additionally, *S. cerevisiae* strains NX01 and NX02 from strong-flavor *Daqu* also exhibited good growth (OD_600_ > 1.5). Strains with OD_600_ > 1.5 were further screened for beer brewing characteristics (see Table S3). In media where maltose serves as the sole carbon source, all tested strains of *S. fibuligera* (G02-G06, Hx01-Hx05) and both S. cerevisiae strains (NX01-NX02) demonstrated robust growth capabilities, with OD_600_ values exceeding 1.5, suggesting their strong potential for wort fermentation applications. Thus, these strains were selected for further experiments. Currently, wild yeasts of the genus *Saccharomycopsis* remain understudied in terms of taxonomy and ecology, necessitating detailed physiological characterization to assess their potential domestication as brewing starters [5].

#### 3.2.2. First Round of Rescreening

**Figure 2:**
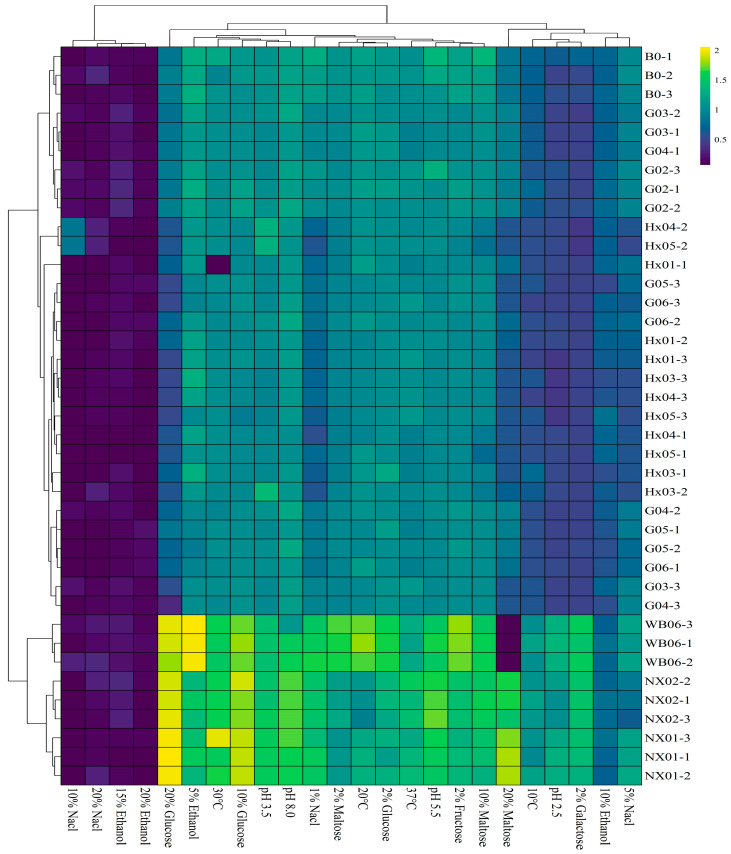
Heatmap of the strains during the first round of rescreening: evaluation of physiological characteristics.

Incubation temperature significantly influenced *S. fibuligera* strain growth, with cultures at 20 °C, 30 °C, and 37 °C exhibiting approximately 50% higher final absorbances compared to those at 10 °C. In contrast to the *S. fibuligera* strains, both screened *S. cerevisiae* strains (NX01 and NX02) showed OD_600_ > 1 at all tested temperatures. Notably, *S. cerevisiae* NX02 showed significantly higher OD_600_ values at 37 °C and 10 °C compared to *S. cerevisiae* NX01 and WB-06 (Figure 2; Table S4). The incubation temperature significantly influenced non-*Saccharomyces* yeast growth. An optimal propagation temperature promotes high-quality biomass production. In this study, *S. fibuligera* strains exhibited higher absorbance at 20 °C and 30 °C than at 10 °C.

pH is a critical factor that influences microorganism growth, as certain yeasts can alter the rate of pH change during fermentation [40]. While yeasts can maintain internal pH via cellular buffers, they cannot tolerate all pH ranges [41]. The screened *S. cerevisiae* strains (NX01 and NX02) exhibited robust growth at various pH levels (OD_600_ > 1). At pH 8.0, NX01 and NX02 strains showed higher absorbance (OD_600_ = 1.62 and 1.67, respectively) than WB-06 (OD_600_ = 1.43) (*p* < 0.05). All screened *S. fibuligera* strains survived at pH values of 3.5, 5.0, and 8.0, indicating their potential for beer brewing, as standard wort pH ranges from 5.0 to 5.7 [41]. 

Strong beers are becoming increasingly popular among consumers, especially in China, where there is a preference for beverages with higher alcohol content [42,43]. Osmotic stresses and ethanol are the major limiting factors in brewing strong beer with high-gravity wort [42]. Increasing ethanol levels in the culture medium reduced growth, with significant inhibition above 5% ethanol (OD_600_ < 1). However, all strains studied, regardless of their isolation matrix or genetic group, exhibited good growth on 2% glucose, 2% fructose, and 2% maltose (OD_600_ = 0.90~1.0). In contrast, the same concentration of galactose severely restricted the growth of all screened *S. fibuligera* strains (OD_600_ = 0.47~0.50) (see Figure 2; Table S3). Osmotic pressure tolerance analysis using glucose and maltose indicated that most *S. cerevisiae* (NX01 and NX02) strains (OD_600_ ≥ 1.5) thrived when cultivated in a medium containing 20% maltose and 20% fructose. However, these concentrations of maltose and fructose significantly limited the growth of *S. fibuligera* strains (OD_600_ < 1). Osmotolerance is strain-dependent [44], and the *S. cerevisiae* NX02 strain exhibits the potential to surmount challenges, thriving in media containing 10% and 20% glucose and maltose. Investigating the salt tolerance of yeasts is crucial for industrial fermentations, as it can enhance ethanol production and mitigate the risk of contamination by microorganisms with low halotolerance [45]. The *S. fibuligera* strains G02 and B0 showed moderate growth at 1% and 5% NaCl. The yeast strains G02 and B0 demonstrated sustained growth and halotolerance up to 5% NaCl. However, they failed to survive at higher concentrations of 10% and 20% NaCl. These findings suggest that *S. fibuligera* strains G02 and B0 could be well suited for fermenting Gose-style beers, which intentionally feature elevated salt levels [46].

**Figure 3:**
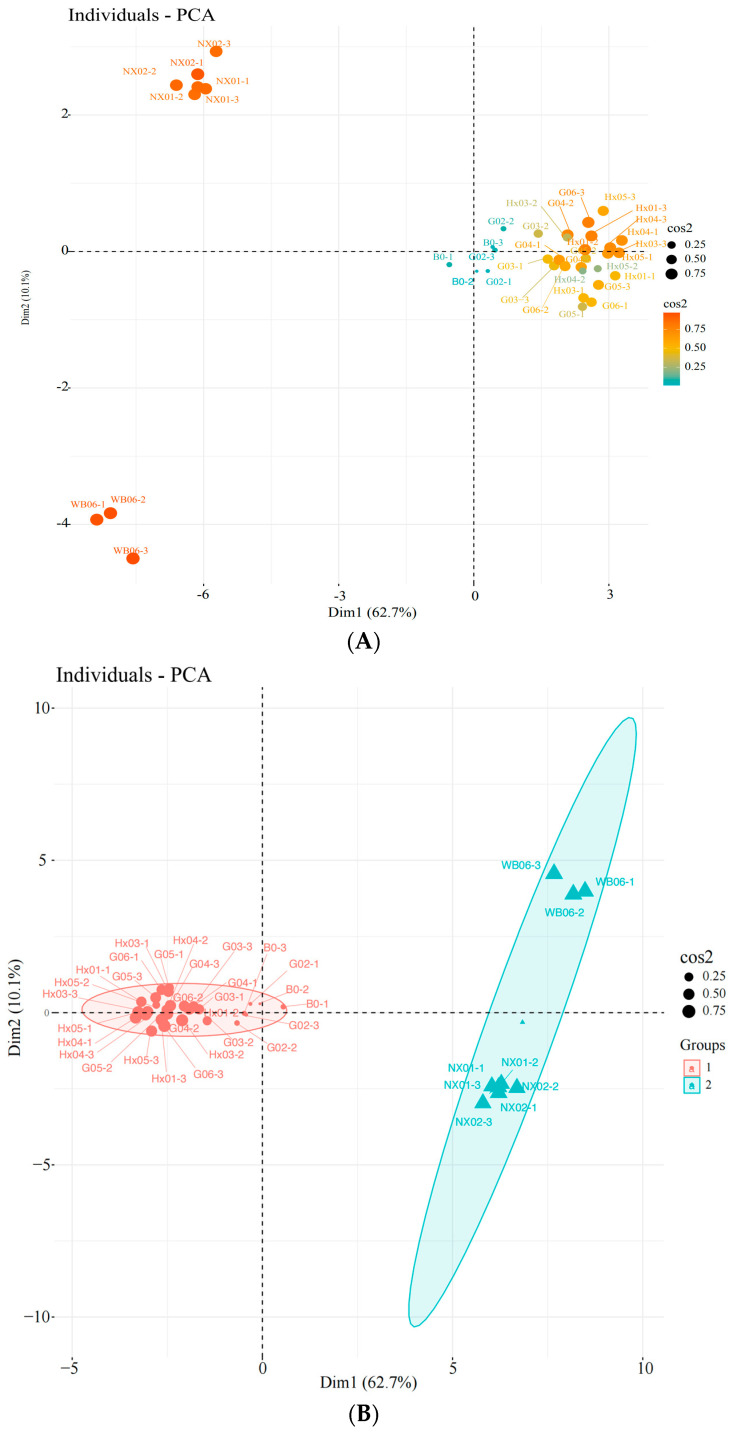

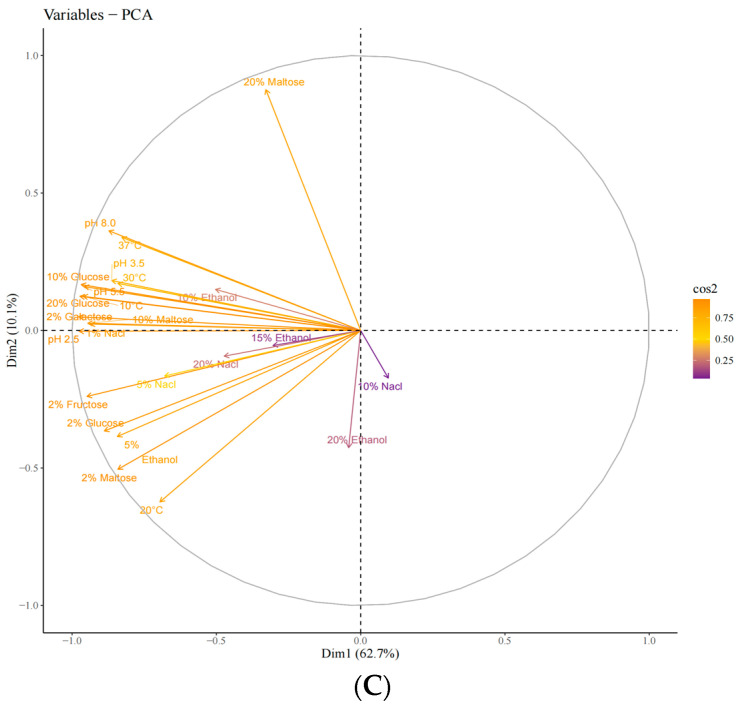
Principal component analysis (PCA) of the strains during the first round of rescreening: evaluation of physiological characteristics. (**A**) Individuals: PCA; (**B**) individuals: PCA; (**C**) variables: PCA.

Unsupervised principal component analysis (PCA) was used to assess the variability between sample groups, subgroups, and within groups for the screened yeasts. PC1 and PC2 contributed 62.7% and 10.1%, respectively, indicating clear separation between sample groups, no outliers, and good clustering by yeast type (Figure 3A–C). Based on the score plot, samples G02, G03, and G04, which exhibited better physiological traits, were selected for further sensory evaluation.

#### 3.2.3. Second Round of Rescreening: Beer Fermentation Test

**Figure 4:**
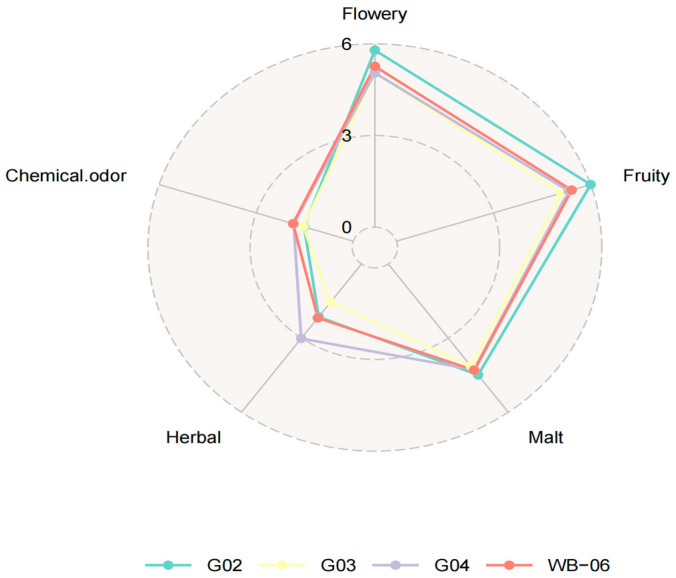
Radar chart of the second rescreening round: beer fermentation test.

Beer fermented with *S. fibuligera* G02 scored higher in sensory analysis for taste complexity, intensity, aroma, acidity, and overall satisfaction, demonstrating its potential as a non-conventional strain for beer production (Figure 4). Therefore, *S. fibuligera* G02 was selected as the preferred non-*Saccharomyces* yeast strain for beer fermentation tests (Tables S5 and S6).

Understanding the potential of the physiological characteristics of *S. fibuligera* strains at multiple levels will facilitate providing an optimal environment for niche adaptation (domestication), promote the ability to address challenges on a large scale, and achieve more predictable fermentation processes. The strain of brewer’s yeast used is a crucial factor that influences the aroma and taste quality of beer, thereby shaping consumers’ perceptions [47]. *S. fibuligera* G02 showed good sensory scores in this study.

### 3.3. Fermentation Optimization of S. fibuligera G02

**Figure 5:**
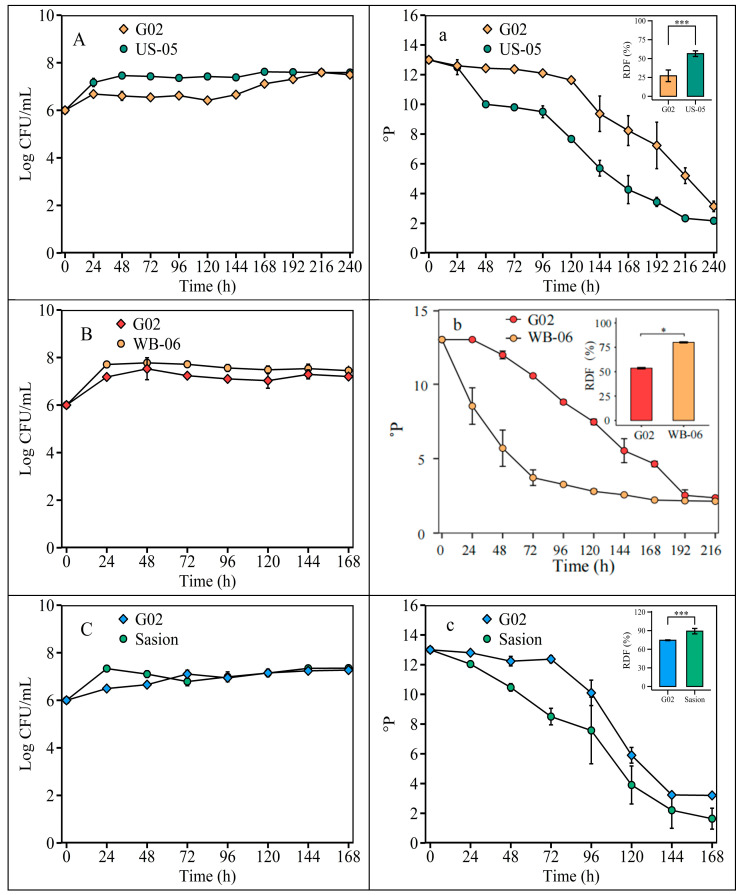
Fermentation temperature optimization. Fermentation is monitored by changes in yeast cell counts (log CFU/mL) in suspension and wort density (^o^P). Note: (**A**) and (**a**), fermentation was at 22 °C, with US-05 as a control strain. (**B**) and (**b**), fermentation was at 26 °C, with WB-06 as the control strain. (**C**) and (**c**), fermentation was at 30 °C, with Saison as the control strain. The error bars represent the standard deviation of the mean. Statistical differences were determined by one-way ANOVA followed by Tukey’s test; * *p* < 0.05 and *** *p* < 0.001.

The ADF for the *S. fibuligera* G02 strain was 27.17 ± 7.68% at 22 °C, 53.58 ± 1.02% at 26 °C, and 74.50 ± 0.63% at 30 °C (Figure 5).

**Figure 6:**
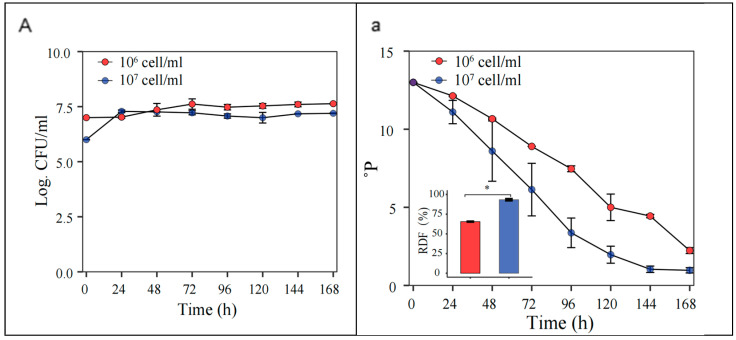
Optimization of initial inoculation rates. Fermentation is monitored by changes in yeast cell counts ((**A**); log CFU/mL) in suspension and wort density ((**a**);^o^P). The error bars represent the standard deviation of the mean. Statistical differences were calculated using Tukey’s test following one-way ANOVA; * *p* < 0.05.

At an initial pitching rate of 1 × 10^7^ cells/mL, the *S. fibuligera* G02 strain showed enhanced fermentability, reaching an ADF of 93 ± 3.0%, compared to 65 ± 1.1% at 1 × 10^6^ cells/mL (Figure 6).

**Figure 7:**
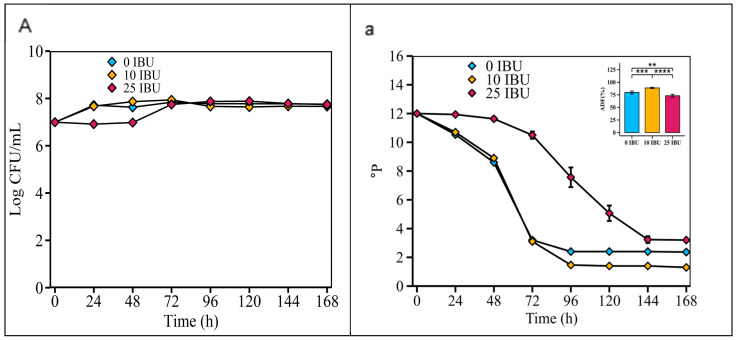
Optimizing initial concentrations of iso-α-acids in wort. Fermentation is monitored by changes in yeast cell counts ((**A**); log CFU/mL) in suspension and wort density ((**a**); ^o^P). The error bars represent the standard deviation of the mean. Statistical differences were calculated using Tukey’s one-way ANOVA; **, adjusted *p* < 0.01; ***, adjusted *p* < 0.001; ****, adjusted *p* < 0.0001.

According to Figure 7, the fermentability of the *S. fibuligera* G02 strain was highest in the 10 IBU wort (88.67 ± 1.21%), significantly higher than in the 0 IBU wort (79.83 ± 2.79%) and the 25 IBU wort (73.25 ± 2.93%).

Monitoring carbohydrate concentrations throughout the fermentation process is crucial for optimizing the efficiency of primary fermentation, which in turn is essential for gaining a comprehensive understanding of yeast carbohydrate consumption kinetics. Sugars in wort are key substrates that are converted by yeast into alcohol and CO_2_ [48]. The ability of yeasts to transport fermentable sugars from the wort into their cells limits the fermentation process [48]. Enhanced fermentation capacity improves flavor and reduces residual sugar, reduces residual sugar, and yields less sweet beer [49]. The fermentation temperature significantly affects microbial growth and product flavor [24,26]. Moderate fermentation at 26 °C balances economic benefits and flavor retention [50]. Thus, the suitable fermentation temperature for *S. fibuligera* G02 beer brewing was 26 °C, and it may be able to ferment ale beer production. Increasing the yeast concentration from 10^6^ to 10^7^ cells/mL accelerated fermentation; thus, an initial yeast inoculation dose of 10^7^ cells/mL was selected as the optimal dose for *S. fibuligera* G02 in this study. Hops are added during wort boiling to impart bitterness and just before or during fermentation to significantly impact yeast fermentation and enhance hoppy flavors and aromas [51]. Hop polyphenols stabilize beer non-biologically, fostering a stable fermentation environment [52]. Excessive hops, owing to the presence of essential oils, can inhibit yeast growth, suggesting that IBU levels in the wort influence the fermentability of brewer’s yeast [53]. Thus, *S. fibuligera* G02 showed optimal fermentability in wort with 10 IBU compared to wort with 25 IBU and 0 IBU (Figure 7). Additionally, IBU values vary depending on the beer type. For example, wheat beer typically has 15–20 IBU, resulting in 15–20 mg of iso-α-acids per liter, while Pilsners range from 30 to 38 IBU, with highly hopped IPAs reaching up to 100 IBU [26]. Thus, *S. fibuligera* G02 may excel at fermenting lightly hopped (10 IBU) wheat beerworts.

**Figure 8:**
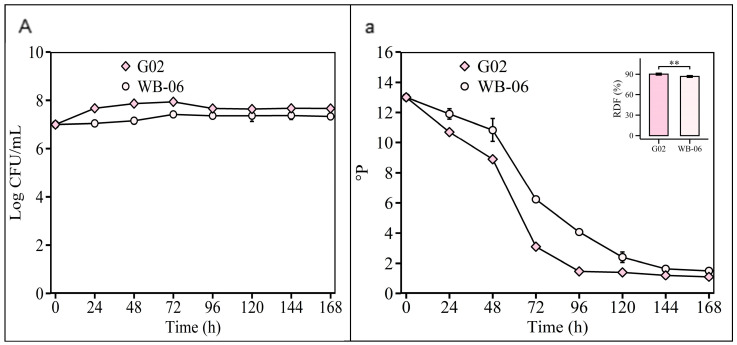
Validation of optimized fermentation parameters. Fermentation is monitored by changes in yeast cell counts ((**A**); log CFU/mL) in suspension and wort density ((**a**); ^o^P). The error bars represent the standard deviation of the mean. Statistical differences were calculated using Tukey’s one-way ANOVA; **, adjusted *p* < 0.01.

In the Section 2.2.4 experiment, the validation of the optimized fermentation parameters showed that both *S. fibuligera* G02 and *S. cerevisiae* WB-06 reached high levels of fermentability (ADF ≥ 85%) at 7 days (Figure 8). The alcohol content of *S. fibuligera* G02 was 4.98 ± 0.03 *v*/*v*, consistent with conventional beers (Table S7). Ethanol, which is crucial for beer flavor, depends on the substrate and yeast alcohol tolerance [54]. However, non-*Saccharomyces* yeasts typically exhibit a lower alcohol production efficiency [12,55]. *S. fibuligera* G02 produces 4.98% alcohol, reflecting its fermentation capabilities, and meets the commercial beer standards (5% *v*/*v* alcohol content) (Table S5), in contrast to the limitations of other non-*Saccharomyces* strains [12,13,45,56].

### 3.4. Micro-Fermentation

**Figure 9:**
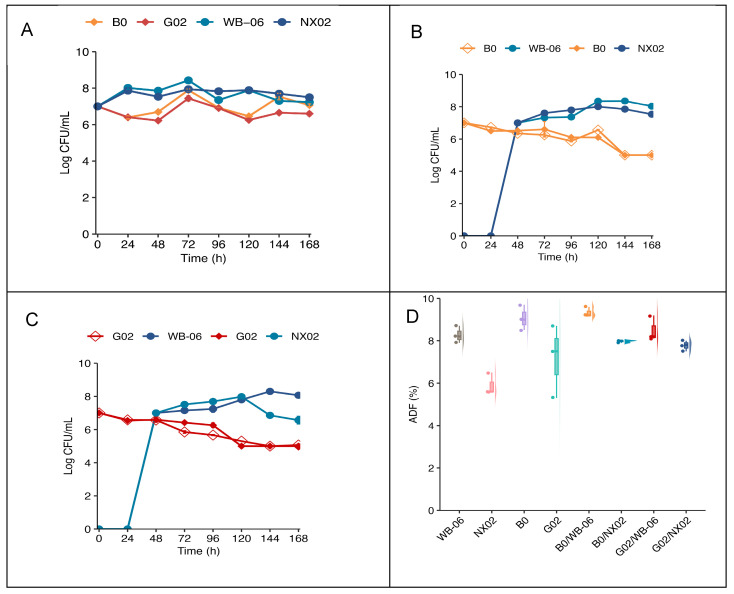
Lab-scale fermentation. (**A**) Yeast cell counts during single-strain fermentation. (**B**,**C**) Yeast cell counts during dual-strain fermentation. (**D**) Box plot showing the ADF (%) of lab-scale fermentation. The error bars represent the standard deviation of the mean.

After establishing the optimal fermentation conditions (Section 3.3), single-strain and dual-strain sequential fermentations were performed using the artisanal wort on the lab scale. The evolution of yeast populations during fermentation is presented in Figure 9.

#### 3.4.1. Yeast Growth During Fermentation

Figure 9 indicates the growth of the four non-*Saccharomyces* strains and *Saccharomyces* during single-strain and dual-strain sequential fermentation. In pure fermentation, both *S. cerevisiae* strains exhibited similar growth patterns, increasing by approximately one log unit over 72 h, followed by a slight decrease. In contrast, *S. fibuligera* G02 and B0 exhibited a growth lag during the first 48 h, reaching their maximum biomass at approximately 72 h, with an increase of less than 1 log (Figure 9A). Distinct cell types were identified based on morphological characteristics (Figures S5–S7). During dual-strain sequential fermentation, the number of viable *S. fibuligera* cells steadily decreased during the first two days. After the addition of *S. cerevisiae* strains, different trends emerged. *S. cerevisiae* counts increased, whereas *S. fibuligera* density declined. After 7 d, *S. cerevisiae* cell concentration rose by one log unit, generally exceeding that of *S. fibuligera* in each group (Figure 9B,C). *S. fibuligera* G02 exhibited a significantly higher final fermentation degree (ADF = 90 ± 0.5%) in single-strain fermentation, but dual-strain sequential fermentation indicated no significant differences in fermentation degrees (*p* < 0.05) (Figure 9D; Table S8).

The yeast fermentation performance, as assessed by growth rate, indicated a lag phase in single-strain fermentations of *S. fibuligera* G02 and *S. fibuligera* B0 for 48 h due to stress responses in *Saccharomycopsis* [57]. *Saccharomycopsis* requires an additional 48 h for fermentation recovery compared to *Saccharomyces* yeast, and this affects fermentation initiation. To mitigate competitive inhibition and enhance fermentation performance, *Saccharomyces* yeast strains were added 48 h after initial fermentation for dual-strain sequential beer fermentation [14]. During dual-strain sequential fermentation, the viable suspended cell concentrations of *S. fibuligera* G02 in the broth steadily decreased (Figure 9) due to yeast competition, oxygen limitation, and ethanol toxicity [58]. The experimental results indicated that *S. fibuligera* G02 retained 10% of the viable suspended cells at the end of the primary fermentation (day 7), maintaining stable metabolic activity. Thus, *S. fibuligera* G02 maintains stable metabolic activity during dual-strain sequential fermentation for an extended period [34]. Increasing the inoculation ratio of *Saccharomycopsis* strains in future mixed fermentations may enhance fermentation performance and beer flavor quality.

#### 3.4.2. Main Volatile Compounds

Beers from G02/WB-06 dual-strain sequential fermentation exhibited higher concentrations of isoamyl alcohol, phenyl ethyl alcohol, isoamyl acetate, and 4VG compared to those of G02 single-strain fermentation. Ethyl acetate and isoamyl acetate concentrations in G02/NX02 dual-strain sequential fermentation were 4.8-fold and 7.9-fold higher, respectively, than those in G02 single-strain fermentation (*p* < 0.05). In B0/WB-06 dual-strain sequential fermentation, aroma compounds such as isoamyl alcohol, isoamyl acetate, and ethyl decanoate were significantly higher than those in B0 single-strain fermentation. The concentration of ethyl caproate in the B0/NX02 dual-strain sequential fermentation (1.42 mg L^−1^) was significantly higher than that in the B0 single-strain fermentation (*p* < 0.05). Finally, 4-EG production was detected in all dual-strain sequential fermentation groups; however, the levels were significantly lower than those observed in G02 single-strain fermentation experiments. Additionally, 4-EG was exclusively detected in the single-strain fermentations of G02 and B0, with concentrations of 1.40 mg/L and 0.46 mg/L, respectively. Notably, G02 exhibited significantly higher levels of 4-EG compared to B0 (Table 2).

Beer, chocolate, soy sauce, and other fermented products derive flavors from microbial fermentation [59]. Recently, non-*Saccharomyces* yeasts have been indicated to produce enzymes that convert raw materials into flavor compounds such as higher alcohols, esters, and phenolic compounds [60,61]. These compounds enrich beer flavor and nutritional value and are crucial for improving beer quality due to their sensory impact and low thresholds [62,63]. Previous studies have investigated the fermentation of *S. fibuligera* G02 for beer production and its mechanisms of flavor formation [26]. In G02/WB-06 sequential mixed fermentation, isoamyl alcohol and phenylethanol levels were significantly increased (Table 2), indicating that mixed fermentation enhanced alcohol production through the synergistic effects of the yeast strains. Isoamyl alcohol is a key flavor compound, and 2-phenylethanol acts as a marker for fermentation parameters [63]. This study identified 2-phenylethanol as the most significant metabolite produced by *S. fibuligera*, and this is consistent with the findings of Lee et al. [56]. The G02/NX02 sequential mixed fermentation produced significantly higher concentrations of ethyl acetate and isoamyl acetate compared to other groups (Table 2). This is likely attributed to secreted enzymes present in *S. fibuligera* G02, including α-L-arabinofuranosidase, β-glucosidase, polygalacturonase, cellulase, and protease. Furthermore, beer serves as a natural source of antioxidants, particularly polyphenols, which are mainly derived from malts, hops, cereals, and other ingredients [5]. The presence of 4-ethyl guaiacol (4EG), known for its ‘spicy’, ‘clove-like’, and ‘vanilla’ notes, is sought after in specialty beers [64]. 4EG is also found in *Baijiu*, wine, coffee, and soy sauce, where it is prized for its smoky aroma in a variety of food products [65]. Research indicates that 4EG has antioxidant and cytoprotective properties [66]. In beer, the concentration of 4EG is determined by hydroxycinnamic acid precursors and the metabolic capabilities of yeast [14]. In this study, 4EG was exclusively produced by *S. fibuligera* strains (B0 or G02) (Table 2).

#### 3.4.3. Calculation of Relative Odor Activity Value (rOAVs) and PCA

**Figure 10:**
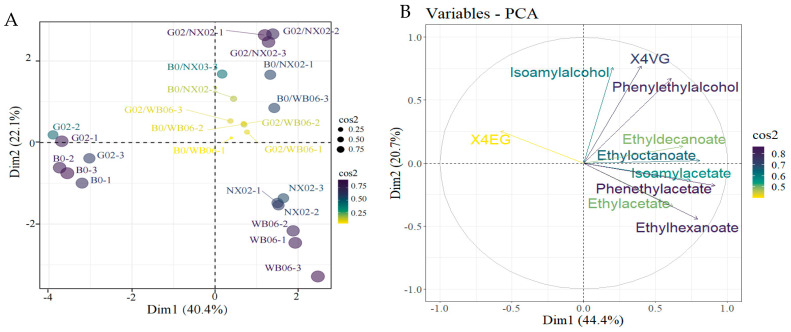
Principal component analysis (PCA) of beer flavor compound composition in lab-scale fermentation. (**A**) Individuals: PCA; (**B**) variables: PCA.

To analyze the differences between beer samples fermented with various non-*Saccharomyces* yeast genera, volatile compounds identified by HS-SPME-GC-MS were analyzed using PCA. Figure 10 illustrates the flavor compound composition of beers brewed with different aroma-generating yeast strains. The loading scores for each flavor compound were calculated (Figure 10). Two principal components (PC1 and PC2) were extracted, accounting for 65.1% of the total variability (PC1: 44.4%; PC2: 20.7%). Most volatiles (44.4%) were located in PC1, closely associated with WB-06, NX02, G02/WB-06, G02/NX02, B0/WB-06, and B0/NX02. The G02 and B0 single-strain fermentation samples were clearly separated from the other groups, indicating significant differences in their aromatic characteristics. High absolute values were observed for isoamyl alcohol, ß-phenylethanol, ethyl acetate, isoamyl acetate, ethyl hexanoate, ethyl caprylate, ethyl phenylacetate, ethyl caprate, and 4VG, contributing substantially to the distinction of samples in PC1. Additionally, 4EG significantly contributed to the separation of samples along PC2.

rOAVs (relative odor activity values), defined as the ratio of a compound’s concentration to its detection threshold, are used to evaluate aroma contribution [67]. Compounds with rOAVs of over 1.0 significantly contribute to flavor [68]. In this study, the production of 2-phenyl ethanol, isoamyl acetate, ethyl caprylate, and 4-ethyl guaiacol exceeded the sensory detection thresholds in the fermentation group inoculated with *Saccharomycopsis fibuligera* strains. Additionally, ethyl hexanoate, phenyl ethyl acetate, and ethyl decanoate exhibited higher rOAVs in dual-strain sequential fermentation than in pure fermentation, indicating that dual-strain fermentation enhanced the production of these esters (Table 3).

Chemometric methods are indispensable for uncovering trends and correlations within complex datasets. Principal component analysis (PCA) is particularly effective in establishing significant links among strains, variables, and values, as well as in correlating sensory attributes with chemical compositions [73]. This study utilized PCA to compare beers fermented with non-*Saccharomyces* yeasts, uncovering that the floral notes in G02/WB-06 samples were attributable to 2-phenyl-1-ethanol, which contributes to the beer’s flavor intensity [74]. The G02/NX02 samples showed elevated levels of ethyl acetate and isoamyl acetate, which are known to enhance the ‘tropical fruit’ aroma profile [75]. B0/WB-06 samples were found to produce more 4VG, whereas G02 single-strain fermentation resulted in higher 4EG levels. Consequently, the inclusion of *S. fibuligera* G02 not only boosts polyphenol production but also enriches the complexity of the volatile compound profile [76].

## 4. Conclusions

In this work, *Saccharomycopsis fibuligera* yeast strains, which can produce diverse flavors during alcoholic beverage fermentation, were isolated from traditional Baijiu fermentation environments. Through fermentative activity analysis of these yeast isolates, we selected *S. fibuligera* G02 for further fermentation characterization under conditions simulating typical beer wort fermentation. To our knowledge, this study provides the first systematic evaluation of how *S. fibuligera* G02—a newly isolated strain with brewing potential—affects beer aroma profiles when co-inoculated with conventional *Saccharomyces* brewer’s yeast, using chemometric approaches. The findings show that 2-phenyl-1-ethanolethyl acetate, isoamyl acetate, and 4EG are the key aroma components of selected *S. fibuligera* G02. Beyond its impact on volatile organic compound production, this study also demonstrated the feasibility of modulating the flavor profile in finished beer through controlled inoculation strategies involving *S. fibuligera* G02 and different *Saccharomyces* brewer’s yeast strains. Future research will focus on developing more sophisticated and tailored fermentation processes. Systematic studies, a comprehensive knowledge database, and AI-based predictive models will serve as essential tools for guiding fermentation processes to achieve specific outcomes, such as improved flavor, texture, nutritional value, and health benefits.

## Figures and Tables

**Table 1:** BlastN results were used to identify wild yeasts isolated from fermented grain and *Daqu* samples.

Strain	Top Hit	Identity	NCBI Accession n.	Query Cover	Origin
G02	*Saccharomycopsis fibuligera*	100%	KX904348.1	100%	Sauce-flavor fermented grain
G03	*Saccharomycopsis fibuligera*	100%	MG518196.1	100%	Sauce-flavor fermented grain
G04	*Saccharomycopsis fibuligera*	100%	KX376260.1	100%	Sauce-flavor fermented grain
G05	*Saccharomycopsis fibuligera*	100%	OR786903.1	100%	Sauce-flavor fermented grain
G06	*Saccharomycopsis fibuligera*	100%	OR786905.1	100%	Sauce-flavor fermented grain
Hd01	*Saccharomycopsis fibuligera*	100%	MK722491.1	100%	Sauce-flavor *Daqu*
Hd02	*Saccharomycopsis fibuligera*	100%	MK722478.1	100%	Sauce-flavor *Daqu*
Hd03	*Saccharomycopsis fibuligera*	100%	MK722491.1	100%	Sauce-flavor *Daqu*
Hd04	*Saccharomycopsis fibuligera*	100%	MK722491.1	100%	Sauce-flavor *Daqu*
Hd05	*Saccharomycopsis fibuligera*	100%	MK373304.1	100%	Sauce-flavor *Daqu*
Hx01	*Saccharomycopsis fibuligera*	100%	MN648845.1	100%	Light-flavor *Daqu*
Hx02	*Saccharomycopsis fibuligera*	100%	KF717372.1	100%	Light-flavor *Daqu*
Hx03	*Saccharomycopsis fibuligera*	100%	KY705007.1	100%	Light-flavor *Daqu*
Hx04	*Saccharomycopsis fibuligera*	100%	JX6457191.1	100%	Light-flavor *Daqu*
Hx05	*Saccharomycopsis fibuligera*	100%	MN497048.1	100%	Light-flavor *Daqu*
Hx06	*Saccharomycopsis fibuligera*	100%	KU956955.1	100%	Light-flavor *Daqu*
Hh01	*Saccharomycopsis fibuligera*	100%	KY705007.1	100%	Light-flavor *Daqu*
Hh02	*Saccharomycopsis fibuligera*	100%	JX645719.1	100%	Light-flavor *Daqu*
Hh03	*Saccharomycopsis fibuligera*	100%	MT577806.1	100%	Light-flavor *Daqu*
Hh04	*Saccharomycopsis fibuligera*	100%	MK497048.1	100%	Light-flavor *Daqu*
Hh05	*Saccharomycopsis fibuligera*	100%	CP012823.1	100%	Light-flavor *Daqu*
Hh06	*Saccharomycopsis fibuligera*	100%	CP095750.1	100%	Light-flavor *Daqu*
NX01	*Saccharomyces cerevisiae*	100%	KY109416.1	100%	Strong-flavor *Daqu*
NX02	*Saccharomyces cerevisiae*	100%	KY441458.1	100%	Strong-flavor *Daqu*

**Table 2:** Aroma compounds produced during fermentation.

NO. ^1^	Aroma Compounds (mg /L ^2^)									
	Isoamyl Alcohol	2-Phenylethyl Alcohol	Ethyl Acetate	Isoamyl Acetate	Ethyl Hexanoate	Ethyl Caprylate	Phenylethyl Acetate	Ethyl Decanoate	4VG	4EG
WB-06	7.72 ± 0.47 ^ab^	10.86 ± 2.03 ^ab^	1.58 ± 0.61 ^c^	1.48 ± 0.15 ^b^	0.85 ± 0.23 ^bc^	6.77 ± 1.76 ^a^	3.03 ± 0.14 ^c^	3.57 ± 0.7 ^a^	0.92 ± 0.20 ^b^	nd ^b^
NX02	7.26 ± 0.32 ^ab^	9.23 ± 1.02 ^bc^	1.11 ± 0.72 ^cd^	1.90 ± 0.17 ^b^	0.91 ± 0.11 ^b^	4.66 ± 0.43 ^b^	4.38 ± 0.23 ^a^	3.13 ± 0.21 ^b^	ND ^c^	nd ^b^
B0	4.05 ± 0.87 ^b^	6.10 ± 2.29 ^c^	ND ^d^	0.36 ± 0.02 ^c^	ND ^d^	0.43 ± 0.34 ^d^	0.22 ± 0.02 ^d^	0.01 ± 0.00 ^d^	ND ^c^	0.46 ± 0.01 ^b^
G02	8.94 ± 0.49 ^ab^	5.10 ± 2.18 ^c^	0.67 ± 0.00 ^d^	0.36 ± 0.01 ^d^	ND ^d^	0.54 ± 0.32 ^d^	0.36 ± 0.12 ^d^	0.01 ± 0.01 ^e^	ND ^c^	1.40 ± 0.13 ^a^
B0/WB-06	8.99 ± 1.23 ^ab^	9.95 ± 1.19 ^b^	2.26 ± 0.74 ^b^	2.11 ± 0.37 ^ab^	0.64 ± 0.19 ^c^	2.30 ± 0.64 ^c^	2.95 ± 0.53 ^c^	0.71 ± 0.27 ^cd^	1.15 ± 0.24 ^a^	0.45 ± 0.02 ^b^
B0/NX02	5.89 ± 0.96 ^b^	6.58 ± 0.04 ^c^	2.45 ± 0.34 ^b^	1.59 ± 1.40 ^b^	1.42 ± 0.01 ^a^	2.87 ± 0.96 ^c^	3.58 ± 0.77 ^b^	0.73 ± 0.33 ^cd^	ND ^c^	0.38 ± 0.02 ^b^
G02/WB-06	10.21 ± 0.67 ^a^	12.67 ± 0.97 ^a^	1.32 ± 0.06 ^cd^	1.96 ± 0.14 ^ab^	0.50 ± 0.03 ^c^	1.93 ± 0.13 ^c^	3.38 ± 0.01 ^bc^	0.88 ± 0.05 ^c^	1.01 ± 0.20 ^ab^	0.40 ± 0.06 ^b^
G02/NX02	6.61 ± 0.67 ^b^	7.32 ± 0.53 ^c^	3.21 ± 0.67 ^a^	2.86 ± 0.15 ^a^	0.96 ± 0.18 ^b^	2.89 ± 0.57 ^c^	3.07 ± 1.98 ^c^	0.57 ± 0.03 ^d^	0 ^c^	0.40 ± 0.01 ^b^

Note: “^1^” represents the abbreviations of the single-strain and dual-strain sequential fermentation groups. *S. cerevisiae* WB-06 is abbreviated as WB-06, *S. cerevisiae* NX02 as NX02, *S. fibuligera* B0 is designated as B0, and *S. fibuligera* G02 as G02. “^2^” represents the quantified flavor compounds expressed as the mean ± standard deviation from parallel experiments. Mean values in the same column with the same letters are not significantly different at the 95% confidence level (Tukey’s test). “ND” indicates compounds that were not detected.

**Table 3:** Relative odor activity value (rOAVs) during fermentation.

Compounds	Sensory Threshold (mg L^−1^)	Odor Descriptor	rOAVs							
			WB-06	NX02	B0	G02	B0 /WB-06	B0 /NX02	G02 /WB-06	G02 /NX02
Isoamyl alcohol	50 [6]	whisky	<1	<1	<1	<1	<1	<1	<1	<1
2-Phenylethy alcohol	0.06 [6]	Rose	>1	>1	>1	>1	>1	>1	>1	>1
Ethyl acetate	7.5 [6]	Fruity	<1	<1	ND	<1	<1	<1	<1	<1
Isoamyl acetate	0.0025 [6]	Banana	>1	>1	>1	>1	>1	>1	>1	>1
Ethyl hexanoate	0.0015 [69]	Pineapple	>1	>1	ND	ND	>1	>1	>1	>1
Ethyl caprylate	0.005 [70]	Fruity	>1	>1	>1	>1	>1	>1	>1	>1
Phenylethyl acetate	0.25 [71]	Rose	>1	<1	<1	<1	>1	>1	>1	>1
Ethyl decanoate	0.2 [72]	Fruity, sweaty	>1	>1	<1	<1	>1	>1	>1	>1
4VG	0.30 [72]	clove	<1	ND	ND	ND	>1	ND	>1	>1
4EG	0.13 [72]	clove	ND	ND	>1	>1	>1	>1	>1	>1

Values represent the means from three independent replicates. ND, non-detected compounds.

## Data Availability

The original contributions presented in the study are included in the article/Supplementary Material, further inquiries can be directed to the corresponding author.

## Supplementary Materials

The following supporting information can be downloaded at: https://www.mdpi.com/article/10.3390/foods14081395/s1.

## Abbreviations

The following abbreviations are used in this manuscript:

MDPI	Multidisciplinary Digital Publishing Institute
DOAJ	Directory of Open Access Journals
TLA	Three letter acronym
LD	Linear dichroism
