# Supplementary material for: Effect of Non-Saccharomyces Yeasts Derived from Traditional Fermented Foods on Beer Fermentation Characteristics and Flavor Profiles
Source: Foods. 2025 Apr 17;14(8):1395. doi: 10.3390/foods14081395 (PMC12027376; doi:10.3390/foods14081395)
Supplement: Supplementary file 1 [file foods-14-01395-s001.zip › foods-3548678-supplementary.pdf]

Supplementary materials for

Effect of non-*Saccharomyces* Yeasts Derived from Traditional Fermented Foods on Beer Fermentation Characteristics and Flavor Profiles

Yanlin Ma[1], Liangyu Liu[3], Guanhui Hu[1], Shuyi Wang[1], Lei Shan[4], Jingyu Chen[1, 2] *

[1]Key Laboratory of Food Bioengineering (China National Light Industry), College of Food Science and Nutritional Engineering, China Agricultural University, Beijing 100083, China

[2] China Agricultural University-Sichuan Advanced Agricultural & Industrial Institute, Chengdu 611430, China

[3] Kweichow Moutai Co., Ltd., Zun Yi 564501, China

[4] Department of Food Science and Nutrition, University of Minnesota, Saint Paul MN55108, USA

*Corresponding author: chenjy@cau.edu.cn

Figure captions

Figure S1. Sampling location of *Daqu* and fermented grains used in this study.

Figure S2. Phylogenetic tree based on the ITS gene sequence of yeast isolated from *Daqu* and fermented grains.Figure S3. CAD design drawing for European Brewery Convention tube (EBC tube).

Figure S4. Beer fermentation strategies for *Saccharomycopsis fibuligera* G02.

Figure S5. Colony morphologys of *Saccharomycopsis fibuligera* G02 on YPD plate.

Figure S6. Phylogenetic tree analysis of strain *Saccharomycopsis fibuligera* G02.

Figure S7. SEM images of Strain *Saccharomycopsis fibuligera* G02.

Table captions

Table S1. Sample collection location and date.

Table S2. Sensory evaluation of beers - quality descriptors and explanation of the scoring used.

Table S3. Primary screening: growth ability in medium with maltose as the sole carbon source.

Table S4. First round of rescreening: evaluation of physiological characteristics.

Table S5. Physico-chemical indexes of second round of rescreening: beer fermentation test.

Table S6. Second round of rescreening: beer fermentation test

Table S7: Alcohol content of *Saccharomycopsis fibuligera* G02 in the experiment validating optimized fermentation parameters.

Table S8. Apparent fermentation degree (ADF) of Micro-fermentation.

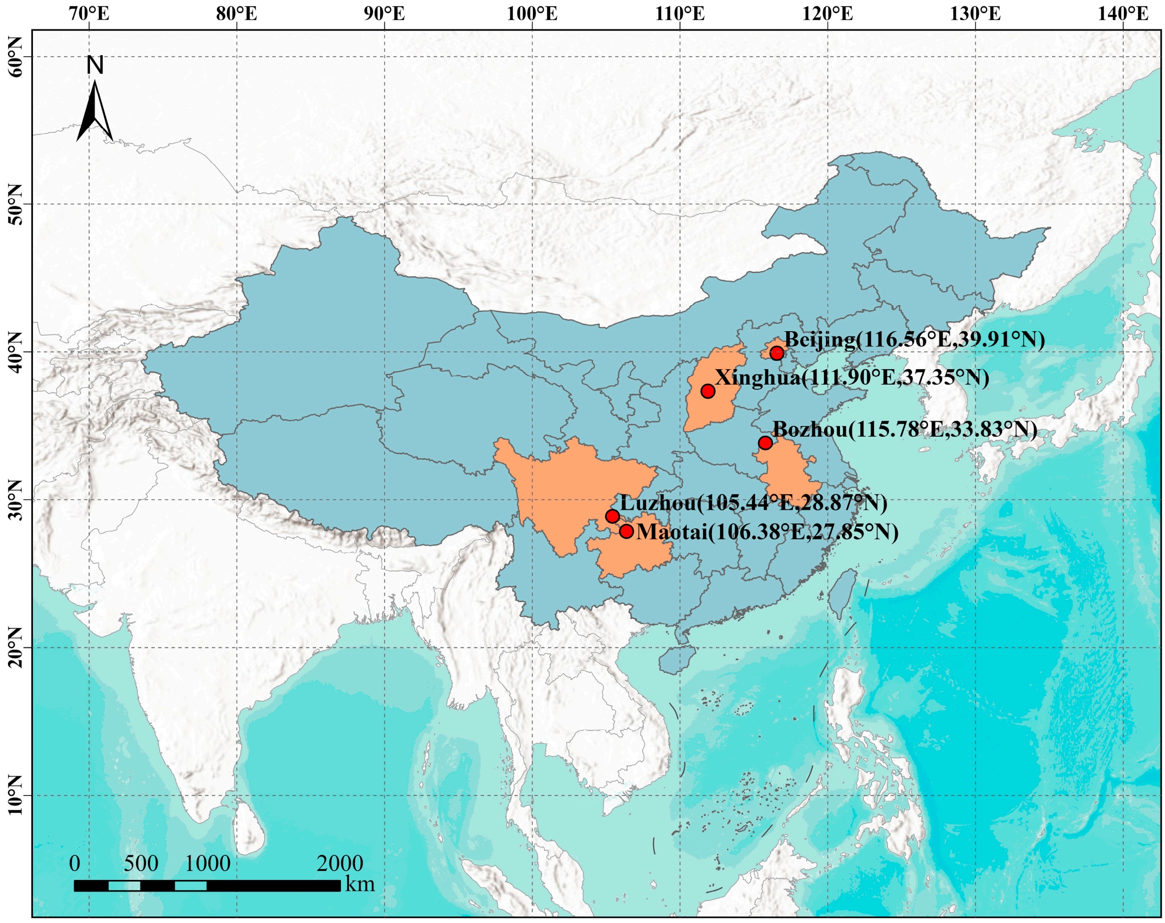

Figure S1. Sampling location of *Daqu* and fermented grains used in this study.

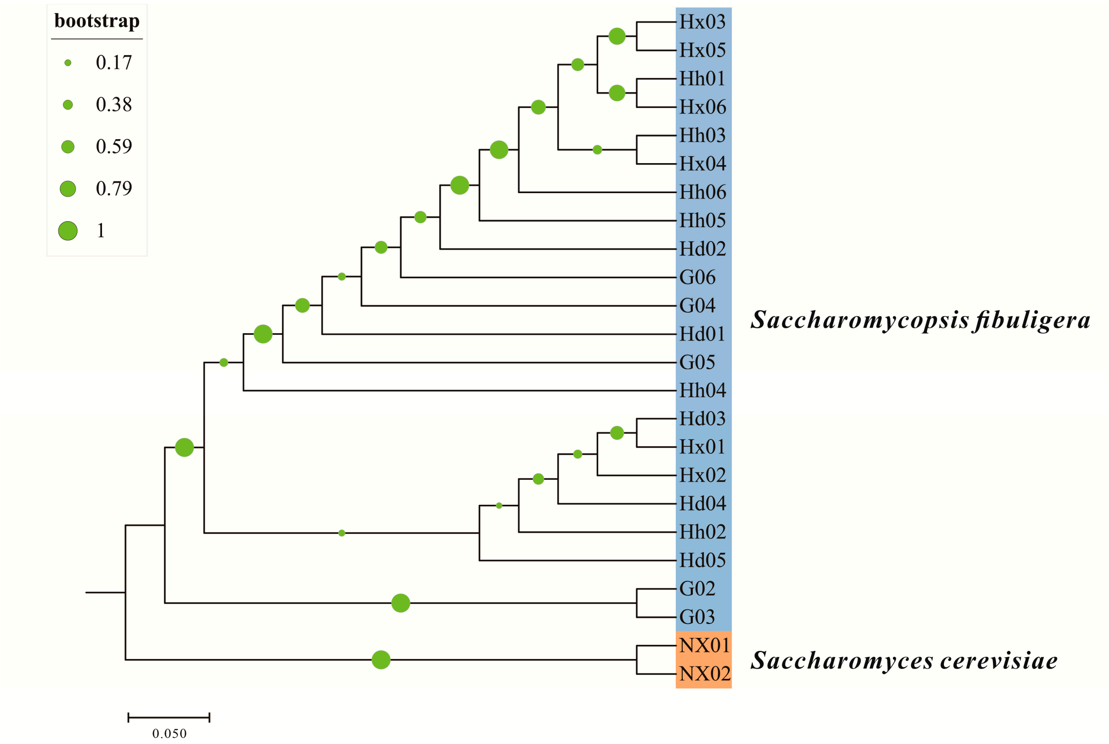

Figure S2. Phylogenetic tree based on the ITS gene sequence of yeast isolated from *Daqu* and fermented grains.

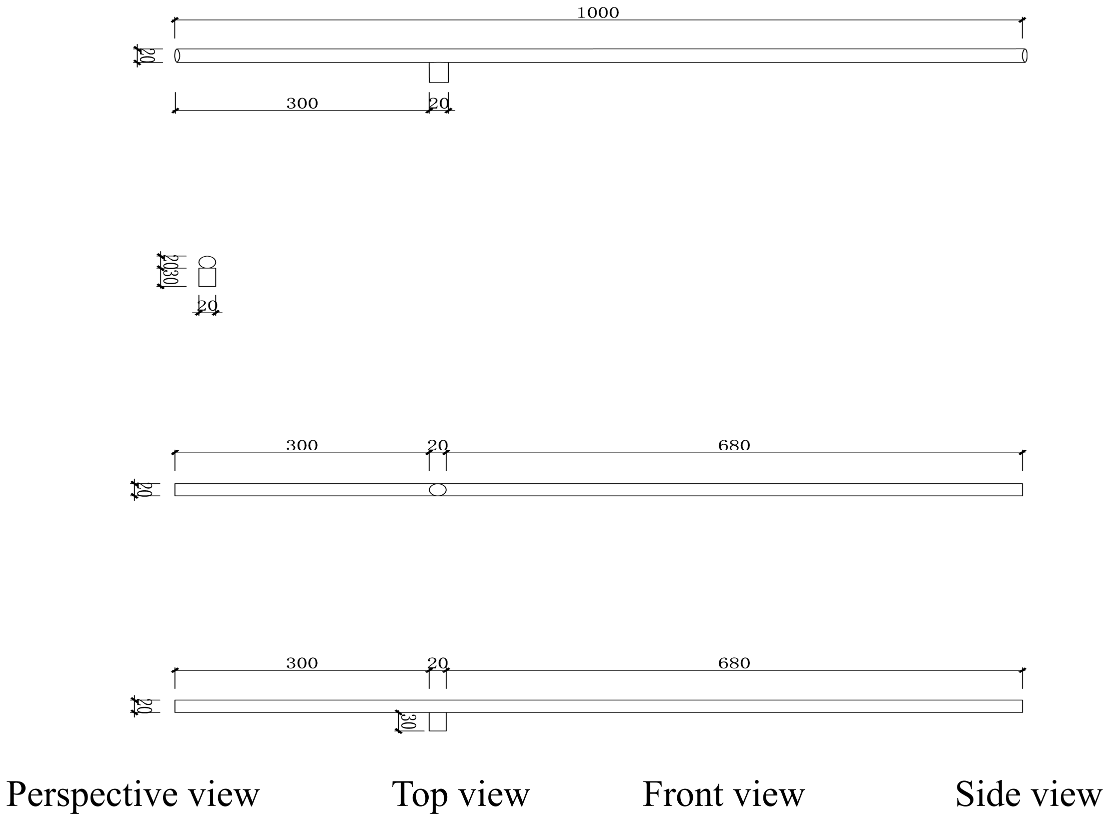

Figure S3. CAD design drawing for European Brewery Convention tube (EBC tube). EBC tube as recommended by the European Brewery Convention (the so-called EBC tube, with dimensions of 2 cm diameter × 100 cm height).

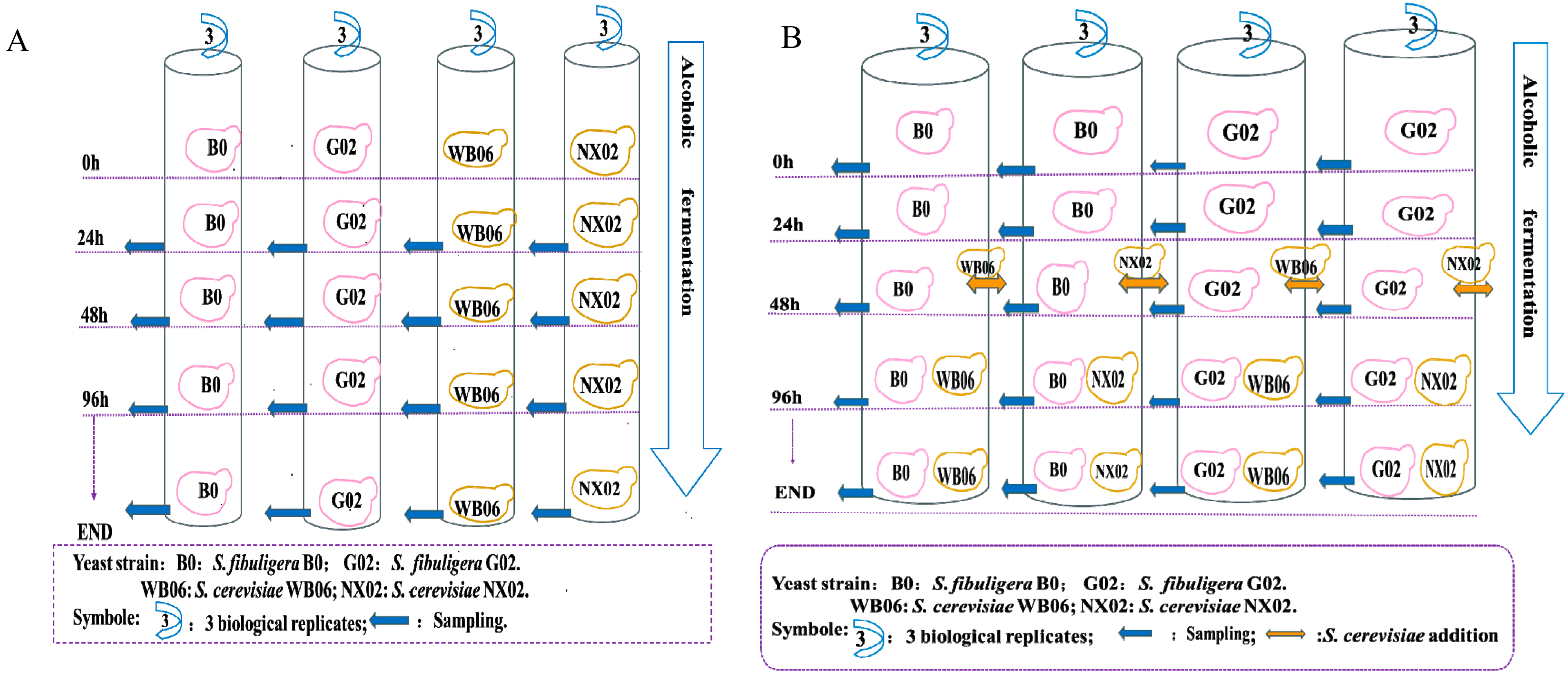

Figure S4. Beer fermentation strategies for *Saccharomycopsis fibuligera* G02. Single-strain fermentation (A) and dual-strain sequential fermentation (B).

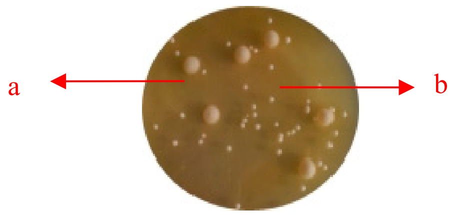

Figure S5. Colony morphologys of *Saccharomycopsis fibuligera* G02 on YPD plate. Note: (a) represents a single colony of the *Saccharomycopsis fibuligera* G02 strain; (b) represents a single colony of the *Saccharomyces cerevisiae* WB-06 strain.

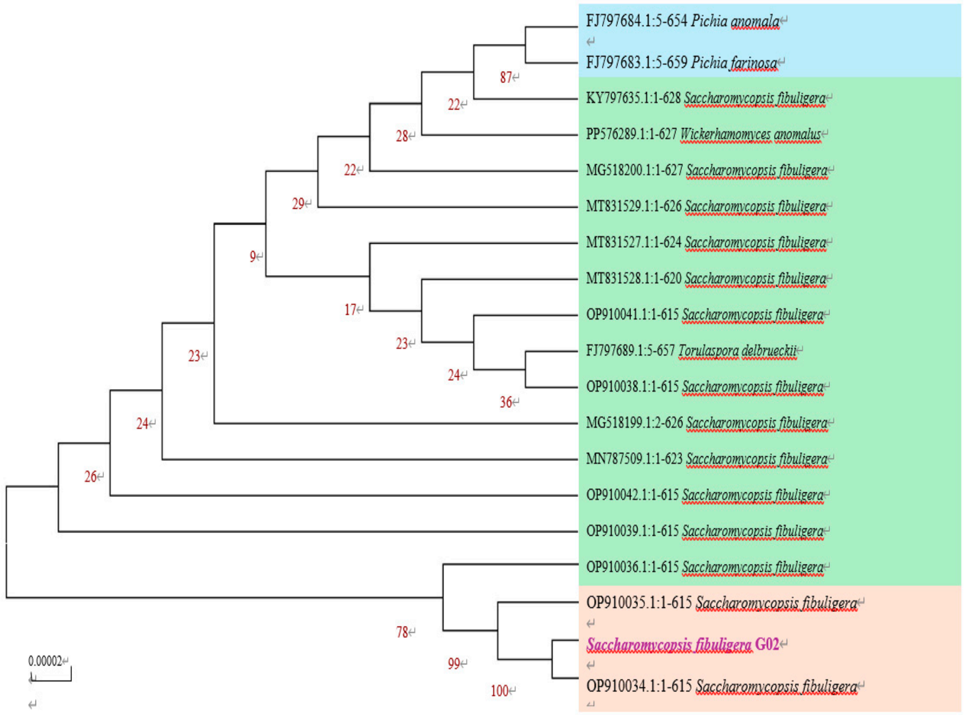

Figure S6. Phylogenetic tree analysis of strain *Saccharomycopsis fibuligera* G02. Note: Homology comparison of the D1/D2 rRNA sequence of *Saccharomycopsis fibuligera* G02, and phylogenetic analysis of the strains using neighbor-joining and bootstrap methods. *Saccharomycopsis fibuligera* G02 was preserved in China General Microbiological Culture Collection Center (CGMCC No. 26747).

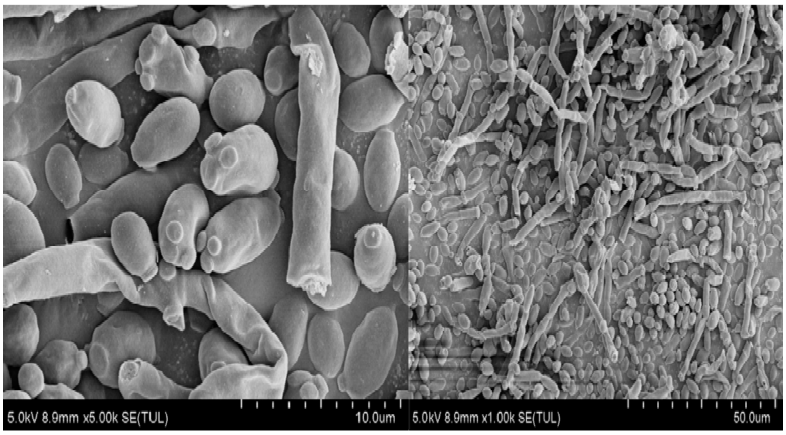

Figure S7. SEM images of Strain *Saccharomycopsis fibuligera* G02.

Table S1. Sample collection location and date.

Sample Number	Sample Type	Collection Location	Collection Time
Hd	Jiangxiang *Daqu*	Maotai Town, Guizhou Province, China	2022
HX	Qingxiang *Daqu*	Xinghua Village, Shanxi Province, China	2022
HH	Qingxiang *Daqu*	Xinghua Village, Shanxi Province, China	2022
NX	Nongxiang *Daqu*	Bozhou City, Anhui Province, China	2022
G0	Jiangxiang fermented grain	Maotai Town, Guizhou Province, China	2022
NX 4~	Nongxiang *Daqu*	Luzhou City, Sichuan Province, China	2022

Note: Among them, three typical geography-dependent aroma types, named Qingxiang (light-flavor type), Nongxiang (strong-flavor type), and Jiangxiang (souce-flavor type).

Table S2. Sensory evaluation of beers - quality descriptors and explanation of the scoring used.

Feature		Score					
Appearance	Clarity	0	Very cloudy;	3	Slightly Cloudy;	6	Clear;
		1	Cloudy;	4	Slightly clerar;	7	Very clear;
		2	Medium Cloudy	5	Medium clerar		
	Color	0	Undesirable;	3	Slightly intense characteristic of the raw materials used	6	Very intense, characteristic of the raw materials used;
		1	very deviating from the natural color of the raw materials;	4	Moderately intense, characteristic of the raw materials used.	7	Intensive, characteristic of the raw materials used
		2	Slightly deviating from the natural color of the raw materials	5	Neutral, characteristic of the raw materials used;		
	Foaminess	0	No foam;	3	Sufficient, persistent,	6	Sufficient, Good persistent, Fine bubble
		1	Insufficient, unstable, low, large bubbles	4	Sufficient; persistent, fine bubble,	7	Sufficient, Good persistent, fine bubble, very abundant, dense
		2	Sufficient, not very persistent, medium bubble	5	Sufficient; persistent, dense		
Malt		0	Not perceptible	3	Medium perceptible	6	Intensely perceptible,

							short-lived
	1	barely perceptible	4	Well perceptible	7	Intensely perceptible; long-lived
	2	very weak perceptible	5	Good perceptible		
Fruity	0	Not perceptible	3	Medium perceptible	6	Intensely perceptible, short-lived
	1	barely perceptible	4	Well perceptible	7	Intensely perceptible; long-lived
	2	very weak perceptible	5	Good perceptible		
Flowery	0	Not perceptible	3	Medium perceptible	6	Intensely perceptible, short-lived
	1	barely perceptible	4	Well perceptible	7	Intensely perceptible; long-lived
	2	very weak perceptible	5	Good perceptible		
Herbal	0	Not perceptible	3	Medium perceptible	6	Intensely perceptible, short-lived
	1	barely perceptible	4	Well perceptible	7	Intensely perceptible; long-lived
	2	very weak perceptible	5	Good perceptible		
Chemical odor	0	Not perceptible	3	Medium perceptible	6	Intensely perceptible, short-lived
	1	barely perceptible	4	Well perceptible	7	Intensely perceptible; long-lived
	2	very weak perceptible	5	Good perceptible		
Mouthfeel	0	Barely perceptible;	4	Intensely perceptible	6	Intensely perceptible

				short-lived		Long-lived<br>Saturation
	1	Well perceptible	5	Intensely perceptible<br>Long-lived	7	Intensely perceptible<br>Long-lived<br>Very good intense
	3	Intensely perceptible	6	Intensely perceptible<br>Long-lived<br>No saturation		
Preference	0		3		6	
	1		4		7	
	2		5			

Table S3. Primary screening: growth ability in medium with maltose as the sole carbon source.

Strain number	OD_{600}
B0	2.19±0.23[a]
G02	2.18±0.19[a]
G03	1.96±0.10[a]
G04	1.98±0.21[a]
G05	1.98±0.14[a]
G06	1.52±0.37[b]
Hd01	1.03±0.10[bd]
Hd02	0.68±0.23[d]
Hd03	1.08±0.23[bd]
Hd04	1.01±0.44[bd]
Hd05	1.08±0.27[bd]
Hx01	1.82±0.25[ab]
Hx02	1.33±0.25[b]
Hx03	2.12±0.07[a]
Hx04	1.76±0.45[ab]

Hx05	1.74±0.01^{ab}
Hx06	0.07±0.05^{e}
Hh01	0.52±0.57^{de}
Hh02	0.53±0.17^{d}
Hh03	0.81±0.31^{d}
Hh04	0.78±0.35^{d}
Hh05	0.51±0.48^{de}
Hh06	1.17±0.35^{bd}
NX01	1.74±0.053^{ab}
NX02	1.75±0.05^{ab}
Blank control	0.09±0.01^{e}

Note: Results represent the mean ± SD for two independent experiments. Mean values in the same row with the same letters are not significantly different at the 95% confidence level (Tukey's test).

Table S4. First round of rescreening: evaluation of physiological characteristics.

	**G02**	**G03**	**G04**	**G05**	**G06**	**Hx01**
10℃	$0.67±0.08^{d}$	$0.63±0.03^{d}$	$0.60±0.05^{de}$	$0.57±0.03^{de}$	$0.54±0.03^{e}$	$0.53±0.02^{e}$
20℃	$1.13±0.04^{cd}$	$1.10±0.07^{cd}$	$1.08±0.06^{cd}$	$1.01±0.01^{d}$	$1.10±0.07^{cd}$	$1.05±0.01^{cd}$
30℃	$1.05±0.01^{b}$	$1.04±0.02^{b}$	$1.02±0.04^{b}$	$1.04±0.01^{b}$	$1.02±0.01^{b}$	$0.72±0.54^{c}$
37℃	$1.09±0.04^{c}$	$1.03±0.10^{c}$	$0.97±0.04^{c}$	$0.99±0.05^{c}$	$1.03±0.10^{c}$	$1.04±0.11^{c}$
pH 2.5	$0.58±0.02^{c}$	$0.50±0.01^{cd}$	$0.49±0.01^{cd}$	$0.49±0.01^{cd}$	$0.51±0.02^{cd}$	$0.48±0.08^{cd}$
pH 3.5	$1.04±0.04^{b}$	$1.03±0.03^{b}$	$1.02±0.04^{b}$	$1.02±0.04^{b}$	$1.04±0.04^{b}$	$1.03±0.03^{b}$
pH 5.5	$1.15±0.17^{b}$	$1.03±0.01^{b}$	$1.04±0.01^{b}$	$1.02±0.07^{b}$	$1.03±0.02^{b}$	$1.03±0.01^{b}$
pH 8.0	$1.20±0.04^{c}$	$1.19±0.05^{c}$	$1.18±0.05^{c}$	$1.16±0.06^{c}$	$1.18±0.05^{c}$	$1.14±0.02^{c}$
5% Ethanol	$1.23±0.05^{cd}$	$1.13±0.08^{d}$	$1.06±0.05^{de}$	$0.96±0.62^{e}$	$1.02±0.08^{de}$	$1.19±0.06^{cd}$
10% Ethanol	$0.77±0.02^{a}$	$0.69±0.01^{ab}$	$0.64±0.05^{bc}$	$0.57±0.04^{c}$	$0.67±0.06^{bc}$	$0.69±0.03^{b}$
15% Ethanol	$0.30±0.02^{a}$	$0.24±0.04^{b}$	$0.18±0.03^{cd}$	$0.14±0.03^{d}$	$0.16±0.03^{cd}$	$0.20±0.02^{c}$
20% Ethanol	$0.13±0.02^{b}$	$0.12±0.02^{b}$	$0.12±0.01^{b}$	$0.18±0.03^{a}$	$0.15±0.04^{ab}$	$0.12±0.01^{b}$
2% Maltose	$1.01±0.02^{d}$	$0.99±0.03^{de}$	$0.97±0.02^{e}$	$1.00±0.24^{de}$	$0.98±0.02^{de}$	$0.97±0.02^{ef}$
2% Glucose	$1.1±0.05^{c}$	$1.09±0.32^{c}$	$1.08±0.26^{c}$	$1.09±0.05^{c}$	$1.05±0.02^{c}$	$1.05±0.02^{c}$
2% Fructose	$1.12±0.03^{d}$	$1.01±0.02^{e}$	$1.01±0.05^{de}$	$1.08±0.04^{cd}$	$1.04±0.04^{de}$	$1.01±0.02^{e}$

2% Galactose	0.52±0.03d	0.47±0.03[d]	0.50±0.01[d]	0.51±0.02[d]	0.50±0.01[d]	0.50±0.02[d]
10% Maltose	1.09±0.01[d]	1.07±0.01[de]	1.06±0.01[de]	1.05±0.01[de]	1.03±0.01[de]	1.00±0.01[e]
10% Glucose	1.16±0.05[c]	1.08±0.02[cd]	1.06±0.04[cd]	1.06±0.04[cd]	1.06±0.04[cd]	1.04±0.05[d]
20% Maltose	0.96±0.03[b]	0.84±0.19[bc]	0.84±0.21[bc]	0.80±0.16[bc]	0.74±0.10[c]	0.70±0.10[c]
20% Glucose	0.89±0.03[b]	0.74±0.15[bc]	0.63±0.27[c]	0.70±0.16[c]	0.66±0.12[c]	0.65±0.11[c]
1% Nacl	1.08±0.04[d]	1.00±0.02[de]	0.94±0.01[e]	0.85±0.04[ef]	0.82±0.03[f]	0.73±0.03[fg]
5% Nacl	0.99±0.01[bc]	0.94±0.05[bc]	0.91±0.47[c]	0.8±0.66[d]	0.74±0.07[de]	0.71±0.07[e]
10% Nacl	0.19±0.02[a]	0.17±0.05[a]	0.13±0.04[a]	0.10±0.01[a]	0.09±0.01[a]	0.11±0.01[a]
20% Nacl	0.15±0.02[a]	0.13±0.02[a]	0.14±0.02[a]	0.1±0.01[a]	0.11±0.02[a]	0.11±0.02[a]

	Hx03	Hx04	Hx05	B0	NX01	NX02	wb06
10°C	0.67±0.08^{d}	0.54±0.03^{e}	0.57±0.03^{de}	0.71±0.02^{d}	1.11±0.10^{b}	1.14±0.05^{a}	1.10±0.01^{c}
20°C	1.08±0.06^{cd}	1.03±0.06^{d}	1.08±0.06	1.17±0.03^{c}	1.29±0.06^{b}	1.03±0.09^{d}	1.73±0.05^{a}
30°C	1.03±0.01^{b}	1.05±0.01^{b}	1.04±0.01^{b}	1.09±0.13^{b}	1.71±0.21^{a}	1.60±0.01^{a}	1.56±0.05^{a}
37°C	0.99±0.06^{c}	1.02±0.09^{c}	1.04±0.09^{c}	1.06±0.03^{c}	1.32±0.09^{b}	1.46±0.11^{a}	1.24±0.04^{b}
pH 2.5	0.48±0.02^{cd}	0.49±0.02^{cd}	0.49±0.04^{cd}	0.57±0.08^{cd}	1.28±0.04^{a}	1.10±0.07^{b}	1.33±0.01^{a}
pH 3.5	1.13±0.23^{b}	1.11±0.20^{b}	1.11±0.21^{b}	1.09±0.02^{b}	1.57±0.01^{a}	1.50±0.06^{a}	1.46±0.04^{a}
pH 5.5	1.02±0.02^{b}	1.02±0.01^{b}	1.01±0.02^{b}	1.22±0.14^{b}	1.57±0.02^{a}	1.67±0.04^{a}	1.57±0.08^{ab}
pH 8.0	1.13±0.01^{c}	1.10±0.06^{c}	1.08±0.05^{c}	1.20±0.01^{c}	1.62±0.04^{a}	1.67±0.01^{a}	1.43±0.30^{b}
5% Ethanol	1.25±0.11^{cd}	1.18±0.06^{cd}	1.07±0.05^{de}	1.30±0.03^{cd}	1.46±0.09^{b}	1.42±0.10^{b}	2.02±0.04^{a}
10% Ethanol	0.60±0.05^{c}	0.66±0.07^{bc}	0.73±0.06^{ab}	0.70±0.03^{ab}	0.73±0.01^{ab}	0.73±0.03^{ab}	0.74±0.07^{ab}
15% Ethanol	0.18±0.03^{cd}	0.12±0.01^{d}	0.11±0.02^{d}	0.15±0.02^{d}	0.12±0.01^{d}	0.28±0.02^{ab}	0.23±0.02^{bc}
20% Ethanol	0.10±0.01^{b}	0.10±0.01^{b}	0.10±0.01^{b}	0.11±0.02^{b}	0.11±0.01^{b}	0.11±0.01^{b}	0.15±0.03^{ab}
2% Maltose	0.96±0.02^{ef}	0.94±0.02^{ef}	0.93±0.01^{f}	1.08±0.02^{c}	1.11±0.01^{c}	1.20±0.04^{b}	1.65±0.02^{a}
2% Glucose	1.11±0.12^{c}	1.05±0.02^{c}	1.05±0.03^{c}	1.09±0.02^{c}	1.27±0.09^{b}	1.33±0.07^{b}	1.62±0.02^{a}
2% Fructose	1.01±0.01^{e}	1.01±0.01^{e}	1.01±0.03^{e}	1.20±0.01^{c}	1.37±0.04^{b}	1.46±0.03^{b}	1.76±0.04^{a}
2% Galactose	0.50±0.01^{d}	0.47±0.05^{d}	0.47±0.05^{d}	0.61±0.10^{c}	1.39±0.06^{b}	1.47±0.02^{ab}	1.54±0.05^{a}
10% Maltose	0.96±0.06^{e}	0.92±0.06^{ef}	0.85±0.12^{f}	1.25±0.10^{c}	1.42±0.08^{b}	1.56±0.04^{a}	1.57±0.04^{a}
10% Glucose	1.00±0.01^{d}	1.00±0.01^{d}	0.96±0.06^{d}	1.11±0.05^{cd}	1.89±0.01^{a}	1.81±0.09^{a}	1.75±0.06^{a}
20% Maltose	0.64±0.29^{c}	0.62±0.001^{c}	0.61±0.19^{c}	0.85±0.03^{bc}	1.80±0.43^{a}	1.59±0.99^{a}	0.78±0.06^{d}
20% Glucose	0.62±0.09^{c}	0.59±0.05^{c}	0.57±0.04^{c}	0.89±0.02^{b}	2.00±0.02^{a}	1.93±0.01^{a}	1.90±0.09^{a}
1% Nacl	0.66±0.05^{g}	0.68±0.10^{g}	0.68±0.07^{g}	1.21±0.06^{c}	1.49±0.09^{ab}	1.47±0.01^{b}	1.57±0.06^{a}
5% Nacl	0.57±0.02^{f}	0.59±0.03^{f}	0.56±0.04^{f}	1.00±0.03^{b}	1.21±0.03^{a}	0.78±0.12^{de}	1.17±0.04^{a}
10% Nacl	0.09±0.06^{a}	0.34±0.03^{a}	0.33±0.12^{a}	0.13±0.060^{a}	0.09±0.01^{a}	0.09±0.06^{a}	0.19±0.09^{a}

20% Nacl	0.20±0.10[a]	0.18±0.10[a]	0.18±0.10[a]	0.22±0.09[a]	0.20±0.19[a]	0.21±0.08[a]	0.25±0.07[a]

Note: Results represent the mean ± SD for two independent experiments. Mean values in the same row with the same letters are not significantly different at the 95% confidence level (Tukey's test).

Table S5. Physico-chemical indexes of second round of rescreening: beer fermentation test

Strains	Beer wort fermentation[a]			
	Final pH	Final gravity	Biofilm[c]	Activity in fermentation[b]
G02	4.78±0.02	2.50±0.15	Yes	Yes
G03	4.78±0.06	2.87±0.2	Yes	Yes
G04	5.25±0.03	3.77 ±0.23	NO	No
WB-06	4.76±0.07	2.80±0.10	NO	Yes

[a] The original gravity of the beer wort was 13°P and initial pH of 5.5.

[b] Activity in fermentation refers to gas production observed through the airlock, bubble formation visible in the liquid culture, or foam at the liquid surface.

[c] Biofilm or "pellicle" is the aggregation of cells through proteins and polysaccharide bonds at the liquid surface.

Table S6. Sensory evaluation of second round of rescreening: beer fermentation test

Group	Flowery	Fruity	Malt	Herbal	Chemical odor
G02	5.85±0.44^{a}	6.05±0.36a	4.58±0.87^{a}	2.13±0.70^{a}	1.52±0.48^{a}
G03	5.13±0.22^{b}	5.20±0.30^{b}	4.15±0.63^{a}	1.65±0.71^{a}	1.68±0.48^{a}
G04	5.17±0.535^{b}	5.38±0.32^{b}	4.31±0.60^{a}	3.02±0.78^{a}	1.83±0.58^{a}
WB-06	5.30±0.446^{b}	5.52±0.38^{b}	4.41±0.56^{a}	2.30±0.88^{a}	1.89±0.77^{a}

Note: Results represent the mean ± SD for two independent experiments. Mean values in the same row with the same letters are not significantly different at the 95% confidence level (Tukey's test).

Table S7: Alcohol Content of *Saccharomycopsis fibuligera* G02 in the experiment validating optimized fermentation parameters.

Group[a]	*S. fibuligera* G02	*S. cerevisiae* WB-06
Alcohol content (V/V)	4.98±0.03[a]	4.75±0.04[b]

Note: Results represent the mean ± SD for two independent experiments. Mean values in the same row with the same letters are not significantly different at the 95% confidence level (Tukey's test).

Table S8. Apparent fermentation degree (ADF) of Micro-fermentation.

NO.[1]	B0	G02	WB06	NX02	B0/WB-06	B0/NX02	G02/WB-06	G02/NX02
ADF[2]（%）	82.73±4.01[a]	90.71±6.12[a]	89.80±8.81[a]	62.71±5.21[b]	89.33±8.32[a]	79.67±5.71[a]	85.22±6.08[a]	77.67±2.52[a]

Note："[1]" represents the abbreviations of the single-strain and dual-strain sequential fermentation groups. *S. cerevisiae* WB-06 is abbreviated as WB-06, *S. cerevisiae* NX02 as NX02, *S. fibuligera* B0 was designated as B0, and *S. fibuligera* G02 as G02."[2]" represents the abbreviations of Apparent fermentation degree (ADF). Statistical differences were calculated through Tukey’s test and one-way ANOVA; Error bars indicate ± SD. Mean values in the same row with the same letters are not significantly different at the 95% confidence level (Tukey's test).
